# Dual modulation of the NLRP3/CASPASE-1 inflammasome pathway and gut microbial metabolism: the antidepressant mechanism of puerarin

**DOI:** 10.3389/fmicb.2026.1907120

**Published:** 2026-08-21

**Authors:** Yu-ting Lu, Yue-yue Chen, Na-na Ding, Ling-wen Meng, Jia-jia Yuan, Ze-xiang Huang, Jing-bo Hu, Jia-xi Tong, Ying-ren Zhang, Nan Nan, Dong-dong Liu, Kai-rui Tang, Xiang Luo, Xia Li, Wen-zhi Hao, Jia-xu Chen

**Affiliations:** 1 Guangzhou Key Laboratory of Formula-Pattern of Traditional Chinese Medicine, Guangdong Second Provincial General Hospital, Integrated Chinese and Western Medicine Postdoctoral Research Station, Jinan University, Guangzhou, China; 2 Guangdong Provincial Hospital of Integrated Traditional Chinese and Western Medicine, Foshan, Guangdong, China; 3 Institute of Biomedical Translational Research, Jinan University, Guangzhou, China; 4 Key Laboratory of Viral Pathogenesis & Infection Prevention and Control, Ministry of Education, School of Medicine, Jinan University, Guangzhou, China; 5 Department of Food Science and Engineering, Jinan University, Guangzhou, China; 6 School of Traditional Chinese Medicine, Beijing University of Chinese Medicine, Beijing, China; 7 School of Basic Medical Sciences, Guangzhou University of Chinese Medicine, Guangzhou, China

**Keywords:** gut-brain axis, microbial metabolism, NLRP3/CASPASE-1 inflammasome, post-stroke depression, puerarin

## Abstract

**Background:**

Post-stroke depression (PSD) is the most common neuropsychiatric complication following stroke. Puerarin (PU), the principal bioactive compound extracted from the medicinal and edible plant *Pueraria lobata*, has shown beneficial therapeutic effects in both depression and stroke. However, the therapeutic effect of PU on PSD and its underlying mechanisms remain unclear. This study investigates the therapeutic efficacy of PU in ameliorating abnormal behaviors in PSD mice and elucidates the roles of intestinal microbiota disorder, intestinal barrier damage, activation of the NLRP3/CASPASE-1 inflammasome in the hippocampus, and dysregulated inflammatory cytokine production in the pathogenesis of PSD.

**Methods:**

To investigate the ameliorative effect of PU on behavioral abnormalities and to clarify the role of intestinal microbiota regulation in the therapeutic effects of PU in PSD mice, various methodologies were employed, including a PSD model, behavioral tests, network pharmacology, hematoxylin-and-eosin staining, ultrastructural morphology, enzyme-linked immunosorbent assay, western blotting, 16S rRNA sequencing, metabolomic analyses, and fecal microbiota transplantation (FMT).

**Results:**

Oral administration of PU could effectively alleviate depressive-like behaviors in PSD mice, repair the damaged colonic mucosa, and increase the expression of occludin and ZO-1. Network pharmacology analysis indicated that the NLRP3/CASPASE-1 inflammasome pathway was a potential therapeutic target of PU, and PU inhibited activation of the hippocampal NLRP3/CASPASE-1 inflammasome. Additionally, PU suppressed pro-inflammatory cytokines in both the hippocampus and serum. PU restored the intestinal microbiota and regulated the microbial metabolism of PSD mice. More importantly, fecal microbiota transplantation from PSD mice reproduced depressive-like behaviors, while fecal microbiota transplantation from PU-treated mice (PU-FMT) prominently relieved depressive-like behaviors in PSD mice.

**Conclusion:**

Our findings indicate that PU reduces depressive-like behaviors in PSD mice by modulating the intestinal microbiota and microbial metabolism and inhibiting the NLRP3/CASPASE-1 inflammasome.

## Graphical abstract

**Figure:**
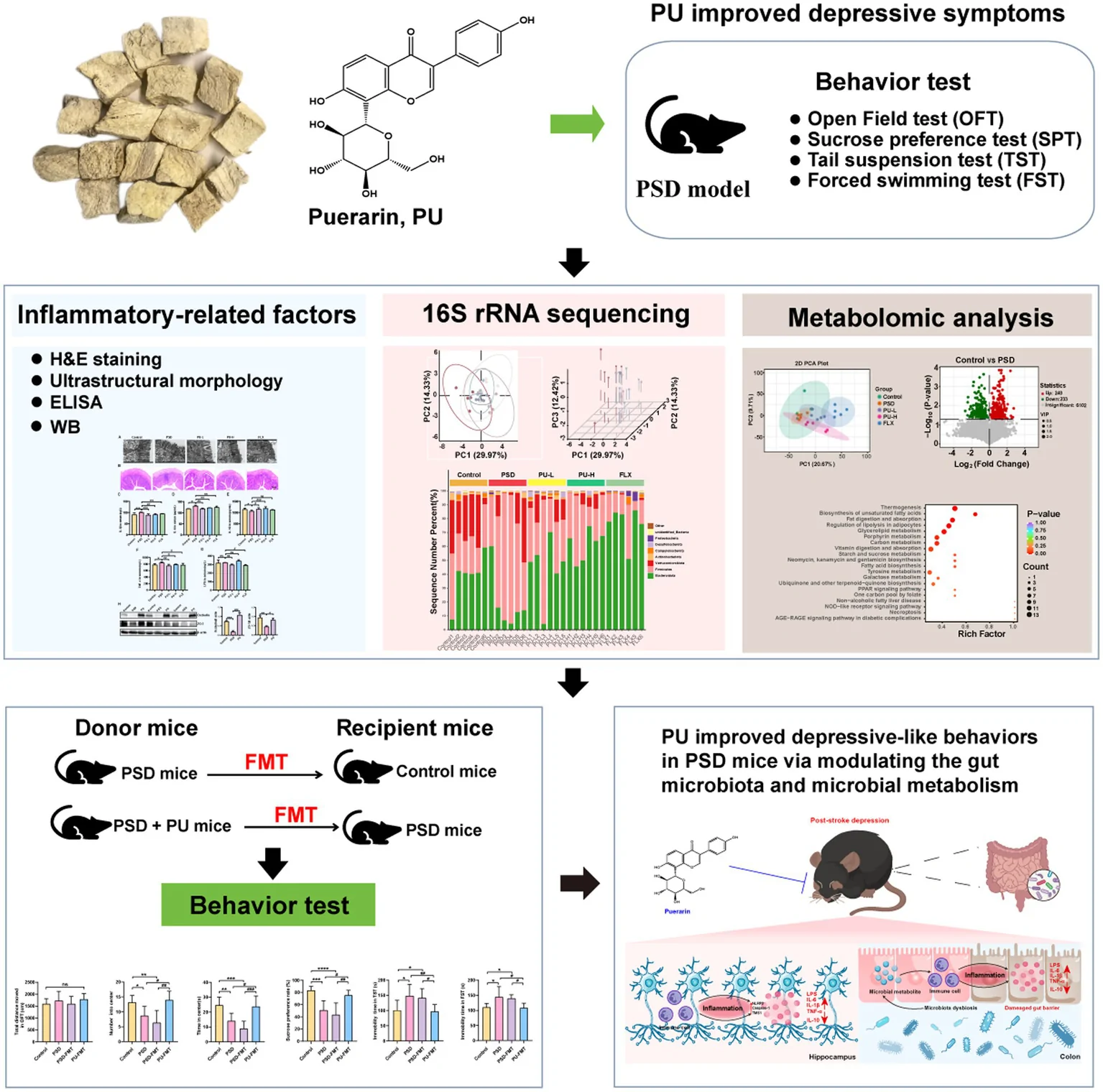
Puerarin improves depressive-like behaviors in post-stroke depression mice by modulating the NLRP3/CASPASE-1 inflammasome and gut microbial metabolism.

## 1 Introduction

Post-stroke depression (PSD) refers to an emotional disorder characterized by depressive symptoms that develop after stroke, affecting approximately one-third of stroke survivors (Medeiros et al., 2020; Castilla-Guerra et al., 2019). Clinically, PSD presents with low mood as its core clinical manifestation, accompanied by numerous pathological changes, including weight loss, insomnia, anxiety, cognitive impairment, and psychomotor agitation (Lam Ching et al., 2023; Wu et al., 2024). In comparison to stroke alone, the presence of depression could exacerbate cognitive impairment and suicidal ideation among stroke patients, which are linked to impaired functionality and elevated mortality rates (Geusgens et al., 2024; Suñer-Soler et al., 2024). With population aging and the growing pressures of social life, the incidence of PSD has increased annually (Shi et al., 2024). To date, PSD has become a major global mental health concern (Le et al., 2024). Unfortunately, experts currently disagree on the best pharmacological approaches for the prevention and treatment of PSD (Gu et al., 2024).

The gut microbiota, the largest microbial community in the human body, operates through the bidirectional gut–brain communication axis, thereby facilitating interactions between the central and peripheral nervous systems (Fabi, 2024). Numerous studies have indicated that disruption of the intestinal microbiota could adversely impact central nervous immune homeostasis via several mechanisms, including inflammatory responses, metabolic alterations, immune system modulation, and neural signaling (Tao et al., 2025; Stolzer et al., 2023). Recent studies have uncovered the pathological association between disruptions in intestinal microbiota and the occurrence of both stroke and depression. For instance, severe cerebral infarction was found to alter the composition of the intestinal microbiota, leading to dysbiosis and triggering a post-stroke immune response. Additionally, it has been demonstrated that fecal microbiota transplantation (FMT) following cerebral infarction treatment leads to a notable enhancement in the prognosis of stroke patients (Hediyal et al., 2024). Similarly, disruptions in intestinal microbiota have been discovered both in depressed patients and animal models of depression (Yuan et al., 2021; Hao et al., 2024). Notably, FMT and probiotic interventions have been found to alleviate depressive-like behaviors, further reinforcing the connection between the onset of depression and intestinal microbiota disturbances (Tian et al., 2022; Kazemi et al., 2019). Intriguingly, current studies have highlighted the crucial role of the intestinal microbiota during the progression of PSD, hinting at the potential for targeting gut microbiota regulation as a therapeutic approach for this comorbidity. Among them, epigenetic and transcriptional alterations triggered by the gut microbiota are regarded as significant contributors to the induction of PSD (Jeong et al., 2023). Regrettably, the scarcity of reports detailing successful interventions using FMT in PSD has thus far prevented the definitive confirmation of the beneficial effects of intestinal microbiota regulation on alleviating PSD symptoms. Although FMT has shown promise in managing standalone cases of stroke or depression, its potential to alleviate the comorbidity associated with PSD remains largely underexplored (Hediyal et al., 2024; Hao et al., 2024).

The basic structure of the NLRP3/CASPASE-1 inflammasome is composed of the receptor protein NLRP3, the apoptosis-associated speck-like protein containing a card (TMS1/ASC), and the effector protein CASPASE-1, which interact to form the NLRP3/CASPASE-1 inflammasome. Recent research has demonstrated an inextricable connection between the intestinal microbiome and the NLRP3/CASPASE-1 inflammasome. On the one hand, the gut microbiome was shown to play a pivotal role in the development and activation of the immune system, promoting the activation of the NLRP3/CASPASE-1 inflammasome; on the other hand, inflammasomes such as the NLRP3/CASPASE-1 inflammasome can trigger the immune system to inhibit the proliferation of pathogens, thereby maintaining the homeostasis of the gut microbiome. At present, the inflammatory response induced by overactivation of the NLRP3/CASPASE-1 inflammasome is related to the pathogenesis of various mental disorders, including depression and anxiety, making it one of the most attractive targets for drug development. Studies have suggested that inhibiting NLRP3/CASPASE-1 inflammasome activity in both the periphery and the central nervous system has antidepressant effects (Du et al., 2016). Moreover, blocking the NLRP3/CASPASE-1 inflammasome signaling cascade can effectively mitigate brain damage, reduce neuroinflammation, and improve neurological recovery (Pan et al., 2025). Numerous studies have indicated that suppressing the NLRP3/CASPASE-1 inflammasome can reduce hippocampal inflammation and effectively improve depressive- and anxiety-like behaviors in PSD mice (Cai et al., 2025). These findings suggest that the inflammation–microbiome axis, formed by the gut microbiome and the NLRP3/CASPASE-1 inflammasome, could serve as an effective therapeutic signaling pathway for PSD.

Puerarin (PU), also known as puerarin flavone, is a prominent bioactive constituent derived from the medicinal and edible plant *Pueraria lobata* (Liu X. et al., 2023a). Renowned for its diverse health benefits, PU exhibits properties such as blood pressure reduction, blood lipid lowering, antitumor, anti-inflammatory activity, and potent antioxidant effects (Liu T. et al., 2023b; Zhou et al., 2013). PU exerted marked antidepressant efficacy in models of chronic mild and unpredictable stress-induced depression, as well as those induced by high-fat diets. The underlying mechanism of these effects has been linked to the ability of PU to regulate brain-derived neurotrophic factor levels and inhibit inflammatory processes, thereby highlighting its promising potential as a psychotherapeutic drug (Gao et al., 2021; Song et al., 2021; Liu Y. et al., 2022). Interestingly, PU has demonstrated remarkable therapeutic efficacy in the treatment of transient cerebral ischemia and stroke, with its mechanism rooted in its ability to mitigate oxidative stress and combat neuroinflammation (He et al., 2017; Kong H. et al., 2019). Regrettably, despite the promising therapeutic potential of PU, there is a conspicuous absence of relevant reports investigating its use specifically for PSD. This scarcity of research has led to a significant knowledge gap regarding the potential of PU to address the comorbidity associated with PSD. Intestinal bacteria/enzymes play a pivotal role in the metabolic absorption and biotransformation of PU (Hao et al., 2022). The activity of the gut microbiota and its enzymes results in the metabolism of PU into metabolites, including daidzein and calyx isoflavones, which exhibit robust pharmacological properties and demonstrate enhanced bioavailability (Hao et al., 2022). However, investigations into PSD have yet to yield any pertinent reports on the ability of PU to modulate the intestinal microbiota.

In brief, this research aims to evaluate the therapeutic potential and possible mechanisms of PU in PSD. By developing a PSD mouse model and employing behavioral assessments, network pharmacology, 16S rRNA sequencing, and metabolomics analyses, we aimed to investigate the impact of PU on behavior, intestinal microbial composition, the NLRP3/CASPASE-1 inflammasome, and metabolic profiles in mice. Subsequently, we used fecal microbiota from PU-treated mice for FMT in PSD mice to elucidate the link between PU-induced behavioral alterations and gut microbiota, thereby identifying novel potential therapeutic targets for PSD.

## 2 Materials and methods

### 2.1 Animals

Seven-week-old SPF male C57BL/6 mice were purchased from Zhuhai Baishitong Biotechnology Co., Ltd. (SYXK (Yue) 2022–0174; SYXK (Yue) 2022–0272). The formal experiment began after the mice had acclimatized for 7 days. The laboratory conditions included a relative humidity of 40%, a room temperature of 21 ± 2 °C, and a 12/12-h light/dark cycle. The whole experiment was conducted in accordance with the guidelines set by the Institutional Animal Ethics Committee (Approval ID: IACUC-20240531-06).

### 2.2 Establishment of animal models

The specific method was based on the literature (Vahid-Ansari et al., 2016) and the protocol developed by our research group (Hou et al., 2020). The PSD model was established by injecting endothelin-1 (ET-1) into the unilateral medial prefrontal cortex, as well as chronic restraint stress (CRS).

Unilateral medial prefrontal cortical stroke: A 1.25% tribromoethanol solution was used to anesthetize the mice (0.02 mL·g^−1^; M2920; Easy Check), which were then fixed in a stereotaxic apparatus (E06181-002; RWD). Subsequently, the hair on the crown of the head was shaved, and the skin was incised along the midline to expose the anterior and posterior fontanelles of each mouse. The attached tissue on the skull was gently wiped away with a cotton swab. The microinjector (7803–05; Hamilton) was fixed to the stereotaxic apparatus, and the skull was leveled with the help of a stereomicroscope. An electric skull drill (0.5 mm HM1005; 07840; RWD) was used to drill two holes on the left side of the skull, using the bregma as the zero coordinate. At point 1, the medial–lateral (ML) coordinate was 0.5 mm; the anterior–posterior (AP) coordinate was 1.5 mm, and the dorsoventral (DV) coordinate was −2.6 mm. At point 2, the ML coordinate was 0.5 mm, the AP coordinate was 2.0 mm, and the DV coordinate was −2.4 mm. After drilling was completed, the injection pump was connected, and ET-1 (GC64457; Glpbio) was injected into the left mPFC at a rate of 100 nL·min^−1^, with 500 nL injected at each point, followed by a 5-min dwell time. Meanwhile, an equal amount of artificial cerebrospinal fluid (PH1851; Phygene) was injected into the Control mice following the same procedures. After the procedure, the scalp wound was sutured using the intermittent suture method, and erythromycin was applied to the wound. After surgery, ear tags were applied to the mice, which were then rewarmed for 3 h using an electric blanket.

*CRS*: One week after the operation, the mice were restrained on a special frame for 6 h/day. The restraint process lasted for 21 days. Mice in the Control group were not restrained but were deprived of food and water during the restraint period of the CRS group.

### 2.3 Behavioral evaluation of depressive-like behaviors

#### 2.3.1 Sucrose preference test (SPT)

The whole experiment included two phases: training and testing. In the training phase, the experimental group was provided with two bottles of 1% sucrose solution for 24 h, then one bottle of purified water and one bottle of 1% sucrose solution for another 24 h, followed by switching the positions of the two bottles for a further 24 h. Finally, the mice were fasted and deprived of water for 24 h. During the testing phase, the experimenter provided one bottle of purified water and one bottle of 1% sucrose solution to the mice and recorded the volumes of sucrose solution and purified water consumed over a 3-h period. Finally, the sucrose preference rate was calculated as follows: sucrose preference rate = sucrose solution intake/(sucrose solution intake + purified water intake).

#### 2.3.2 Open field test (OFT)

This experiment was carried out in an open-field experimental chamber (100 cm × 100 cm × 50 cm) compartmentalized into four compartments. Before the formal experiment, the experimenter placed the mice in a quiet enclosed space for 10 min of acclimatization, then placed the mice in groups of four at the center of the experimental compartment. The camera recorded the activity of the mice for 5 min. Before replacing the next group of mice, 75% alcohol was used to clean the inner wall of the experimental box. The movement trajectory of the mice was tracked using behavioral analysis software (EthoVision XT software analysis system, Noldus Information Technology, Leesburg, VA, USA), and their total movement distance, frequency of entering the central area, and time spent in the central area were analyzed.

#### 2.3.3 Tail suspension test (TST)

The tails of the mice were fixed with adhesive tape, and the mice were suspended from a horizontal pole 50 cm above the bottom of the test chamber, with three mice in each group. The entire testing time was 6 min, and the EthoVision software analysis system was used to record the movement of the mice during the last 4 min. Subsequently, the immobility time of the mice was calculated, which refers to the time during which the mice ceased struggling and remained immobile.

#### 2.3.4 Forced swimming test (FST)

For this experiment, the mice were put into a cylindrical glass water tank (30 cm high, 5 cm in radius) to swim freely, with the water temperature maintained at 23 ± 1 °C, and the duration of the experiment was 6 min. The EthoVision software analysis system was used to record the movements of the mice and to calculate the immobility time during the last 4 min, defined as the time during which the mice remained still and floated on the water surface.

### 2.4 Pharmacological interventions

**Figure 1:**
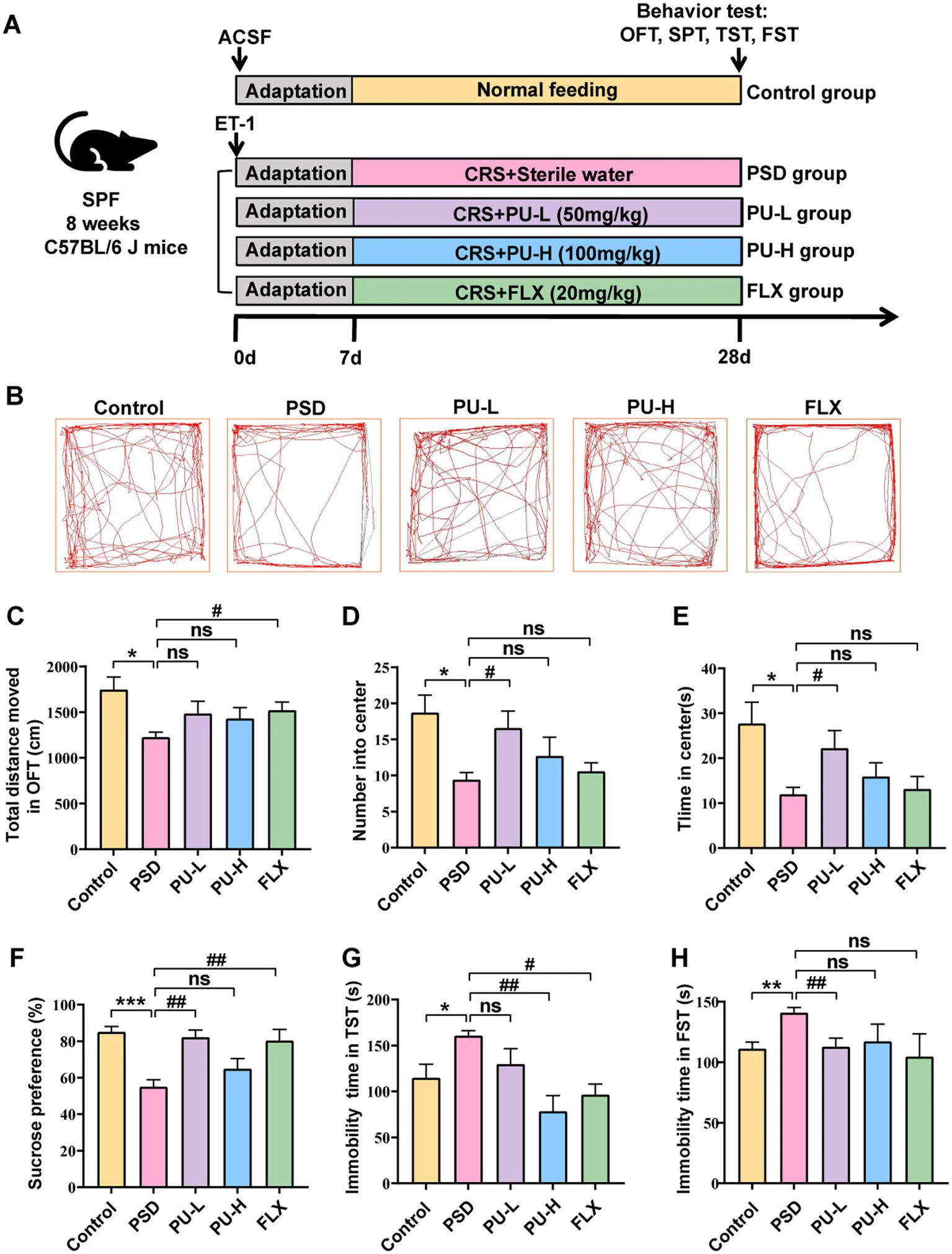
PU significantly improved the depressive-like behaviors of PSD mice. **(A)** Experimental design and procedures. Mice were randomly divided into five groups (*n* = 10 per group). Control group; PSD group (administered sterile water daily); PU-L/H groups (administered PU at 50 or 100 mg·kg^−1^ daily); and FLX group (administered FLX at 20 mg·kg^−1^ daily). **(B)** Representative tracks in the OFT. **(C)** Total distance moved in the OFT. **(D)** Number of entries into the central zone. **(E)** Time spent in the central zone of the OFT. **(F)** Sucrose preference test (SPT). **(G)** Immobility time in the TST. **(H)** Immobility time in the FST. Data are presented as the mean±SEM (*n* = 10 per group). ^*^p < 0.05, ^**^p < 0.01, ^***^p < 0.001 *versus* the Control group; ^#^*p* < 0.05, ^##^*p* < 0.01 *versus* the PSD group.

Experiment 1: After 7 days of acclimatization, 50 C57BL/6 mice were randomly assigned to a PSD model group (*n* = 40) and a Control group (*n* = 10). Animals in the Control group received stereotaxic injection of artificial cerebrospinal fluid into the left mPFC following the same procedure. After the stroke model was constructed by stereotaxic injection of ET-1 into the left mPFC, the modeled mice were randomly divided into the PSD group, puerarin low-dose group (PU-L), puerarin high-dose group (PU-H), and fluoxetine group (FLX), with 10 mice in each group. These groups were subjected to the combined CRS method and treated for 21 days. During this period, the PU-L and PU-H groups received PU solution (B20446; YuanYe) by gavage at doses of 50 mg·kg^−1^ and 100 mg·kg^−1^, respectively. The FLX group received fluoxetine solution (1.84 mg·mL^−1^; 56,296–78-7; Selleck) at a dose of 20 mg·kg^−1^. The PSD and Control groups received equal volumes of sterile water, with a gavage volume of 10 mL·kg^−1^ for all groups (Figure 1A).

**Figure 2:**
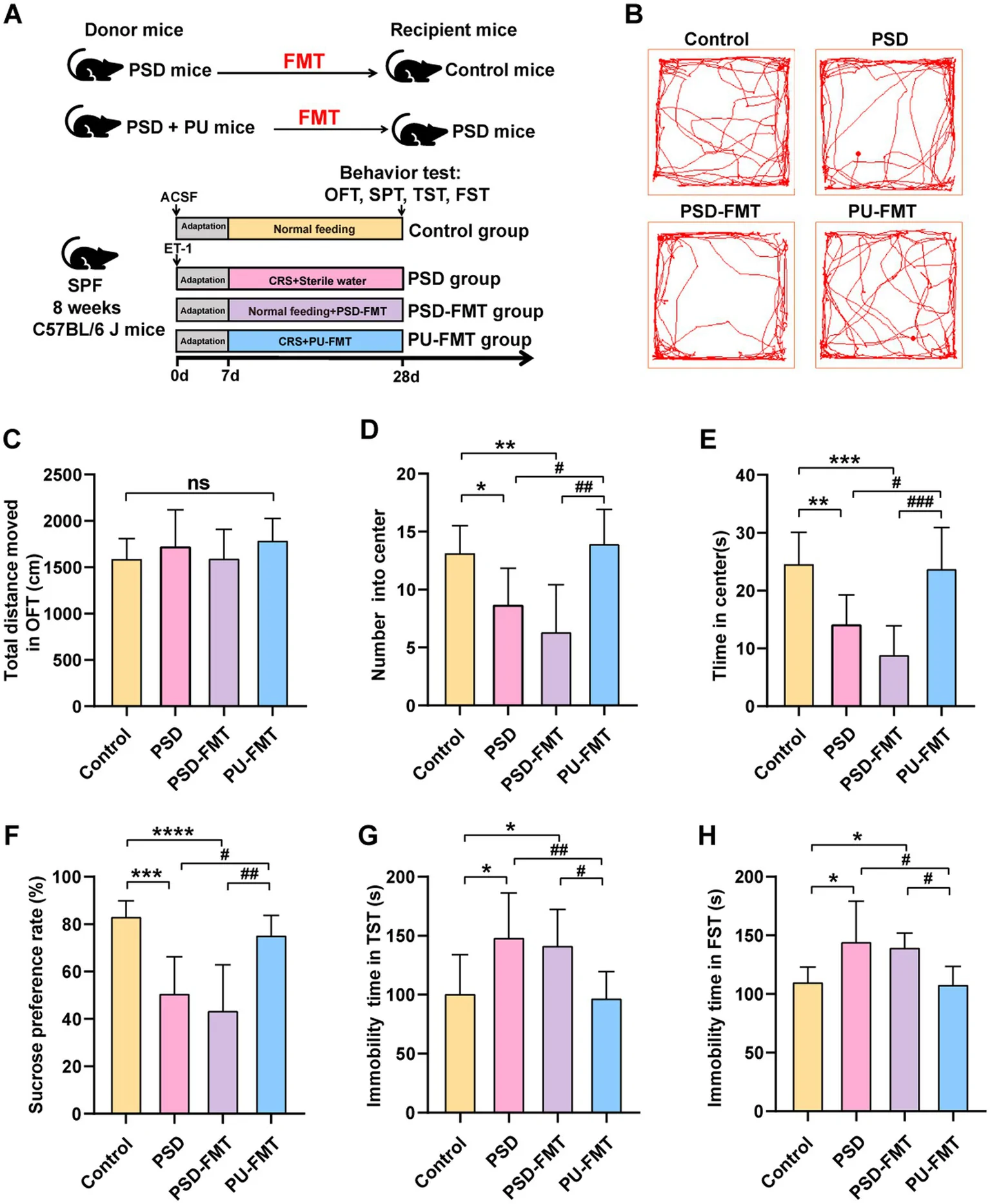
PU attenuates depressive-like behaviors in PSD mice in a gut microbiota-dependent manner. **(A)** Experimental design and procedures. **(B)** Representative tracks in the OFT. **(C)** Total distance moved in the OFT. **(D)** Number of entries into the central zone. **(E)** Time spent in the central zone of the OFT. **(F)** Sucrose preference test (SPT). **(G)** Immobility time in the TST. **(H)** Immobility time in the FST. Data are presented as the mean ± SEM. * *p* < 0.05, ^**^ *p* < 0.01, ^***^p < 0.001.

Experiment 2: After 7 days of acclimatization, 40 C57BL/6 mice were randomly assigned to four groups: Control, PSD, Control+FMT-PSD (PSD-FMT), and PSD + FMT-PU (PU-FMT). The Control group did not receive any special intervention. The PSD group was established by stereotaxic injection of ET-1 into the left mPFC combined with CRS. The solution administered by gavage to the PSD-FMT group was derived from the fecal microbiota solution of the PSD group mice in Experiment 1, and the solution administered by gavage to the PU-FMT group was derived from the fecal microbiota solution of the PU-L group mice in Experiment 1. The fecal microbiota was prepared by aseptically collecting mouse feces, suspending the samples in sterile sodium chloride solution (S917712; Macklin), diluting the suspension to 200 mg feces/2 mL, sequentially filtering it through sterile 200-, 400-, and 800-mesh screens, and vortexing and centrifuging it to obtain the final suspension. The Control and PSD groups received an equal amount of sterile water by gavage at 10 mL·kg^−1^, and the intervention lasted for 21 days (Figure 2A).

### 2.5 Sample collection

On days 22–24 of the formal experiment, behavioral tests were conducted on all mice. After the behavioral tests, fresh fecal samples from the mice were collected aseptically and stored at −80 °C for subsequent 16S rRNA sequencing and metabolomic analyses. The feces of the PSD and PU-L groups were collected aseptically, suspended in 20% glycerol (BL1274A; Biosharp), and stored at −80 °C for subsequent FMT. Next, the mice were anesthetized with 1.25% tribromoethanol solution (0.02 mL·g^−1^; M2920; Easy Check), and orbital blood was collected following enucleation of the left eye. The serum samples were obtained by centrifugation and stored at −80 °C for enzyme-linked immunosorbent assay (ELISA). The hippocampal tissues were also stored at −80 °C and subsequently analyzed by Western blotting (WB) and ELISA to determine the expression of relevant inflammatory markers. A portion of the colon tissue was collected, washed with phosphate-buffered saline, and immersed in 4% paraformaldehyde for HE staining. Another portion of the colon tissue was stored at −80 °C for subsequent WB analysis. The remaining colon tissue was fixed in 2.5% glutaraldehyde (G1102; Solarbio) for ultrastructural observation.

### 2.6 Bioassays

#### 2.6.1 Hematoxylin–eosin staining (HE)

The colonic samples were fixed in 4% paraformaldehyde and processed into paraffin blocks through dehydration and paraffin embedding. Then, 4-μm-thick tissue sections were cut and baked in an oven at 60 °C for 3 h. The sections were dewaxed with xylene, absolute ethanol, and graded ethanol, then stained with hematoxylin for 3–5 min, differentiated in 1% hydrochloric acid alcohol for 2–5 s, counterstained with bluing solution for 2–5 s, and stained with 1% eosin solution for 5 min. The sections were then dehydrated, cleared, and sealed with neutral gum. The tissue sections were scanned and imaged using a pathological section scanner.

#### 2.6.2 Ultrastructural morphology of the colon

Colonic samples were fixed overnight at 4 °C in 2.5% glutaraldehyde solution. The samples were rinsed three times with 1 M phosphate buffer for 15 min each. This was followed by ethanol dehydration, after which the samples were soaked in isoamyl acetate two times for 20 min each. After routine drying treatment, the colonic ultrastructure was observed by electron microscopy (SU8100; Hitachi). Quantitative morphometric analysis of electron micrographs was performed using Fiji (ImageJ), with all images calibrated against the embedded scale bar before parameter measurement.

#### 2.6.3 ELISA

The ELISA detection kits (Cusbio; Wuhan; China) were used to detect the expression of interleukin (IL)-10, IL-1β, IL-6, lipopolysaccharide (LPS), and TNF-*α*. The reagents, samples, and standards were prepared in accordance with the manufacturer’s instructions. The prepared samples and standards were added and incubated for 30 min at 37 °C. The plates were washed five times, and the enzyme-labeled reagent was added; then, the mixture was incubated for 30 min at 37 °C. Subsequently, the plates were washed five times again, and color-substrate solutions A and B were added and incubated for 10 min at 37 °C. Finally, the termination solution was added, and the optical density (OD) values were read within 15 min.

#### 2.6.4 16S rRNA amplicon sequencing

This process mainly included DNA extraction, polymerase chain reaction (PCR) amplification, product purification, library preparation and quality inspection, and Illumina NovaSeq sequencing. The cetyltrimethylammonium bromide (CTAB) method was used to extract genomic DNA from the samples, and agarose gel electrophoresis was performed to determine the DNA concentration and purity. The DNA sample was diluted to 1 ng·μL^−1^ with sterile water and used as the template. Barcode-specific primers, Phusion® High-Fidelity PCR Master Mix with Guanine-Cytosine (GC) Buffer (New England Biolabs), and a high-fidelity DNA polymerase were used to ensure efficient and accurate PCR amplification. The PCR products were identified by 2% agarose gel electrophoresis after thorough mixing. Magnetic beads were employed to purify the qualified PCR products, an enzyme labeling instrument was used to quantify the results, and equal amounts of PCR products were combined based on their concentrations. The Qiagen gel extraction kit was used to recover the target DNA fragments. The library was constructed using the TruSeq® DNA PCR-Free Sample Preparation Kit and qualified by Qubit and Q-PCR analysis. Sequencing was performed on the NovaSeq6000 platform. After obtaining the effective tags, OTU clustering, ASV denoising, species annotation, alpha-diversity analysis, and beta-diversity were performed. The service was provided by Wuhan Maiwei Metabolic Biotechnology Co., Ltd.

#### 2.6.5 Untargeted metabolomics

Metabolomics included collecting and processing samples, extracting metabolites, and detecting and analyzing the metabolomic data. Data analysis mainly included the screening of differential metabolites and metabolic pathway analysis. Complete sample extraction was performed on ice, and the raw data were obtained through LC–MS analysis. The data were preprocessed, including mass spectrometry peak extraction, calibration, and metabolite annotation. Then, data quality control, TIC overlap and correlation analysis of QC samples, CV distribution mapping, and CV filtering were carried out to obtain high-quality data. The data were analyzed by statistical methods such as PCA and cluster analysis.

#### 2.6.6 Western blotting (WB)

Western blotting was used to examine the expression of NLRP3 (1:1000, AB263899, Abcam), TMS1/ASC (1:1000, DF6304, Affinity), and CASPASE-1 (1:1000, 24,232 T, CST) proteins in the hippocampus, as well as occludin (1:1000, 27,260-1-AP, Proteintech) and ZO-1 (1:1000, 21,773-1-AP, Proteintech) proteins in the colon. *β*-actin (1:5000, 66,009-1-Ig, Proteintech) was used as the internal loading control. Hippocampal and colonic tissues were first removed from the −80 °C freezer, weighed, minced, and placed in a pre-chilled homogenizer tube containing protein extraction buffer for total protein extraction (1 mg of tissue per 10 μL of extraction buffer). The tissues were thoroughly ground at low temperature using a high-throughput tissue grinder. After incubation on ice for 20 min, the lysate was pipetted and mixed thoroughly, then the mixture was incubated for another 10 min on ice. The lysates were then centrifuged for 20 min (4 °C, 12000 rpm), and the supernatants were collected. The BCA protein assay kit was used to determine the tissue protein level, and the proteins were separated by electrophoresis for 30 min at 80 V and then for 1 h at 120 V. After electrophoresis, proteins were transferred onto a PVDF membrane, which was blocked with 5% skimmed milk powder for 1 h at room temperature. The primary antibodies were diluted with TBST, and the membranes were cut according to the molecular weights of the target proteins. The primary antibodies were added to the hybridization boxes and incubated at 4 °C overnight. The membranes were washed with TBST for 10 min per wash, three times, after the primary antibodies had been removed. Subsequently, the secondary antibodies were added to the hybridization boxes and incubated for 1 h, followed by washing with TBST. After development, images were captured and stored using the Tanon-5200 analysis system.

#### 2.6.7 Network pharmacology

The active components of PU were obtained from the SwissTargetPrediction.^1^ Putative bioactive ingredients were then mapped to official gene symbols via UniProt,^2^ with the organism restricted to “*Homo sapiens*.” Disease-related targets for“Ischemic Stroke” and “Depression” were collected from OMIM^3^ and GeneCards,^4^ merged in Excel, and deduplicated by Entry ID to generate disease-specific target sets. The Venn tool^5^ was used to create a Venn diagram by importing PU-related targets, depression-related targets, and ischemic stroke-related targets, from which intersecting targets were extracted. A “puerarin–target–disease” network was constructed in Cytoscape 3.9.1,^6^ and key nodes were preliminarily identified based on degree. Common targets of PU and the two diseases were then submitted to STRING,^7^ with the species set to “*Homo sapiens*,” the minimum required interaction score set to the highest confidence (>0.9), and disconnected nodes hidden. The resulting PPI network was imported into Cytoscape to calculate degree, betweenness centrality, and closeness centrality. Finally, Gene Ontology (GO) and Kyoto Encyclopedia of Genes and Genomes (KEGG) pathway enrichment analyses of these targets were performed using the DAVID database,^8^ and the results were visualized.

### 2.7 Statistical analysis

The statistical analysis was performed using SPSS 25.0 software. The statistical methods included the independent sample t-test for two-group comparisons and one-way analysis of variance (one-way ANOVA) followed by Tukey’s Honestly Significant Difference (HSD) *post hoc* test for multiple pairwise comparisons among more than two groups to control the Type I error rate. Statistical significance was considered at *p* < 0.05. Graphs were generated using GraphPad Prism version 7.0 software (La Jolla, USA).

## 3 Results

### 3.1 PU modified the depressive-like behavior of PSD mice

Behavioral tests (SPT, OFT, FST, and TST) were conducted to clarify the effect of PU on depressive-like behaviors of PSD mice. The results of the OFT indicated that, compared with the Control group, the PSD mice had a significant decrease in the total movement distance, the frequency of entering the central region, and the time spent in the central region (*p* < 0.05; *p* < 0.05; *p* < 0.05) (Figures 1B–E). Compared with the PSD group, the frequency of entering the center and the time spent in the center were significantly increased in the PU-L group (*p* < 0.05; *p* < 0.05) (Figures 1D,E), whereas the reduction in total movement distance also showed a reversal trend (Figure 1C). The frequency and time spent in the center, as well as the overall movement distance, of the PU-H group also showed a reversal trend, but without statistical significance (Figures 1C–E). The frequency and time spent in the center, along with the overall movement distance, of the FLX group also showed an increasing trend, with a statistically significant increase in total movement distance (*p* < 0.05) (Figures 1C–E). The SPT results showed that, compared with the Control group, the sucrose preference of the PSD mice was significantly reduced (*p* < 0.001) (Figure 1F). The sucrose preference rates of the PU-L and FLX groups were significantly higher than those of the PSD group (*p* < 0.01; *p* < 0.01). In addition, the PU-H group also showed an increasing trend without statistical significance (Figure 1F). Compared with the Control group, the TST results showed that the immobility time of the PSD mice was significantly prolonged (*p* < 0.05). Compared with the PSD group, the immobility time of the PU-H and FLX groups was significantly shortened (*p* < 0.01; *p* < 0.05), whereas the PU-L group also showed a decreasing trend (Figure 1G). The FST results showed that the immobility time of the PSD mice was significantly longer than that of the Control group (*p* < 0.01). Compared with the PSD group, the immobility time of the PU-L group was significantly shortened (*p* < 0.01) and that of the PU-H and FLX groups tended to decrease, although the differences were not statistically significant (Figure 1H). According to the comprehensive results of behavioral testing, compared with the Control group, the PSD group showed significant changes in depressive-like behaviors, while the PU-L, PU-H, and FLX groups showed significant improvement. Among these, the PU-L group showed the most significant improvement in behavior, suggesting that PU treatment could effectively alleviate the depressive-like behaviors of PSD mice.

### 3.2 PU restored intestinal integrity and alleviated inflammation in PSD mice

**Figure 3:**
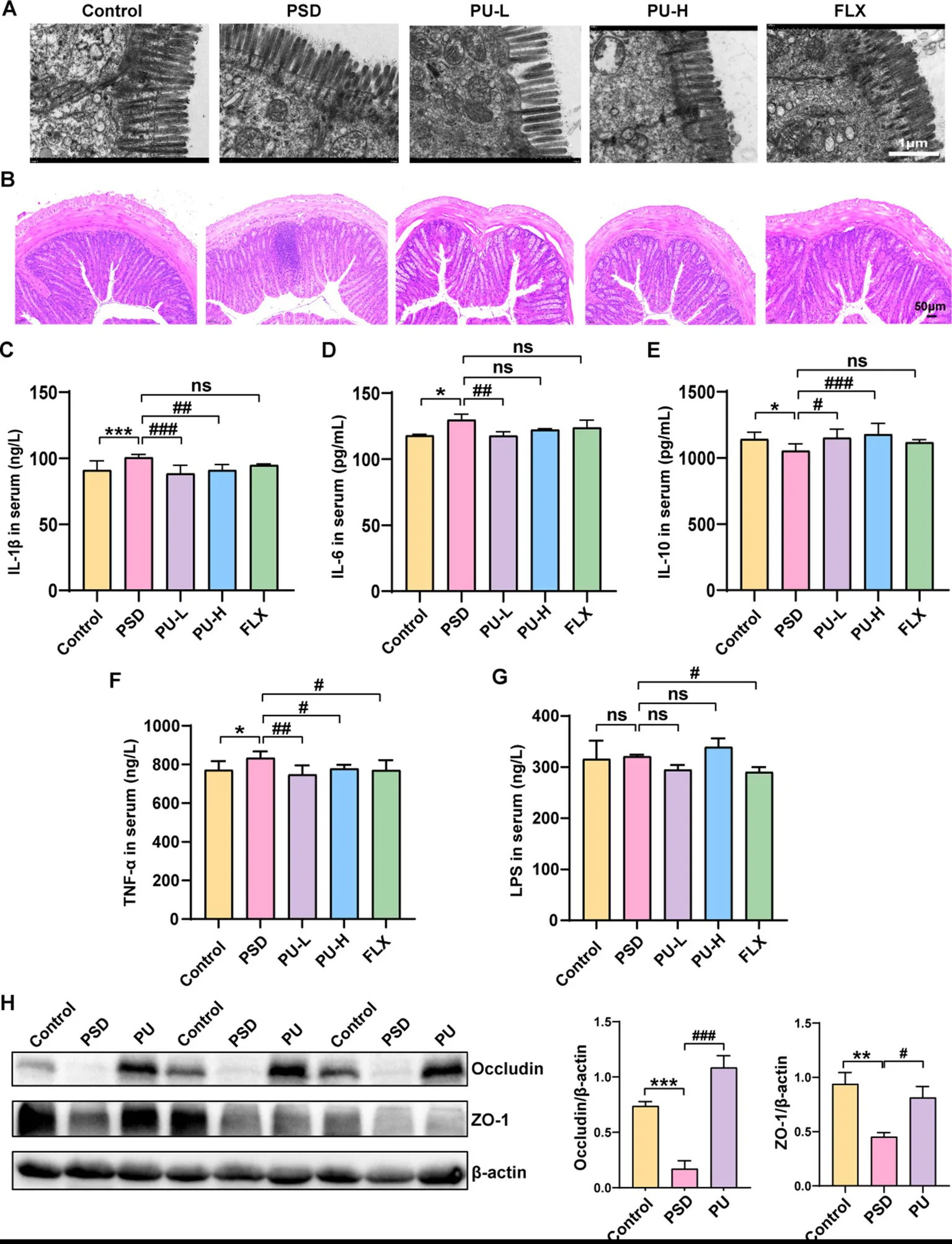
PU repaired the intestinal integrity and alleviated inflammation in PSD mice. **(A)** Transmission electron microscopy. Scale bar = 1 μm. **(B)** H&E staining of the colons. Scale bar = 50 μm. **(C–G)** Serum levels of the inflammatory factors involving IL-1β, IL-6, IL-10, LPS, and TNF-α (*n* = 8 per group). **(H)** Protein expression of occludin and ZO-1 in the colon (*n* = 3 per group). Data are presented as mean ± SEM. ^*^p < 0.05, ^**^p < 0.01, ^***^p < 0.001, ^****^p < 0.0001.

Colon ultrastructure was observed by transmission electron microscopy to evaluate colonic pathological changes in PSD mice and the therapeutic effects of PU and FLX. The observation results indicated that the colon tissue of the Control group was healthy, the intestinal epithelium was intact, and the microvilli were arranged regularly and closely (Figure 3A). In the PSD group, the colon tissue of mice showed obvious pathological changes, including damage or loss of the intestinal epithelial structure, a reduced number of colonic microvilli, and their irregular arrangement (Figure 3A). These observations suggested that the colonic barrier function of the PSD group was damaged, while the colon tissue morphology of the other groups was closer to that of the Control group, and the abnormal arrangement and reduction of microvilli in the PU-L, PU-H, and FLX groups were effectively reversed (Figure 3A). Specifically, the quantitative analysis revealed no notable inter-group differences in microvillus density across all groups. Relative to the Control group, the mean microvillus length was markedly diminished, alongside pronounced increases in microvillus damage and shedding rates within the PSD group (*p <* 0.01; *p <* 0.0001; *p <* 0.001). Compared with the PSD group, the PU-L and FLX groups showed significant increases in average microvillus length (*p <* 0.01; *p <* 0.0001). Furthermore, prominent declines in microvillus damage and shedding rates were observed in the PU-L, PU-H, and FLX groups (*p <* 0.0001; *p <* 0.01; *p <* 0.0001; *p <* 0.001; *p <* 0.01; *p <* 0.001) (attached figure).

The results of HE staining indicated that the colon tissue of the Control group was healthy and intact, without edema or inflammatory cell infiltration (Figure 3B). The colonic epithelial cells of the PSD group showed degeneration, necrosis, and shedding, with diffuse infiltration of inflammatory cells in the interstitium. Compared with the PSD group, the inflammatory cell infiltration in the submucosal layer of the colon was significantly reduced, mucosal integrity was effectively restored, and the number of goblet cells was significantly increased in the PU-L, PU-H, and FLX groups (Figure 3B).

Colonic inflammation and the disruption of barrier function can lead to an increase in serum inflammatory factors. To further explore the changes in systemic inflammation in PSD mice, the production of the anti-inflammatory cytokine IL-10, as well as the pro-inflammatory cytokines IL-6, IL-1β, LPS, and TNF-*α* in the serum of mice, was detected by ELISA. Compared with the Control group, the levels of IL-1β, IL-6, and TNF-α in the serum of PSD mice were significantly increased (*p* < 0.001; *p* < 0.05; *p* < 0.05), and the IL-10 level was significantly decreased (*p* < 0.05) (Figures 3C–E). The serum IL-1β level in the PU-L and PU-H groups was significantly lower than that in the PSD group (*p* < 0.001; *p* < 0.01) (Figure 3C). Compared with the PSD group, the serum IL-6 level in the PU-L group was significantly reduced (*p* < 0.01) (Figure 3D), and the serum IL-10 level in the PU-L and PU-H groups was significantly increased (*p* < 0.05; *p* < 0.001), while the FLX group showed a trend toward reversal without statistical significance (Figure 3E). The serum TNF-α levels in the PU-L and PU-H groups were significantly reduced compared with those in the PSD group (*p* < 0.01; *p* < 0.05; *p* < 0.05) (Figure 3F). Regrettably, serum LPS levels were significantly reduced only in the FLX group (*p* < 0.05), whereas no notable improvements in LPS were observed in either the PU-L or PU-H group (Figure 3G).

The WB results confirmed that the expression levels of the tight-junction proteins occludin and ZO-1 in the colon of PSD mice were significantly decreased compared with those of the Control group (*p* < 0.001; *p* < 0.01), while PU intervention significantly increased the expression of occludin and ZO-1 in the colon of PSD mice (*p* < 0.0001; *p* < 0.05) (Figure 3H). Taken together, these results demonstrated that PU significantly alleviated colonic mucosal damage in PSD mice.

### 3.3 Network pharmacology identified NLRP3 as a potential target of PU for the treatment of PSD mice

**Figure 4:**
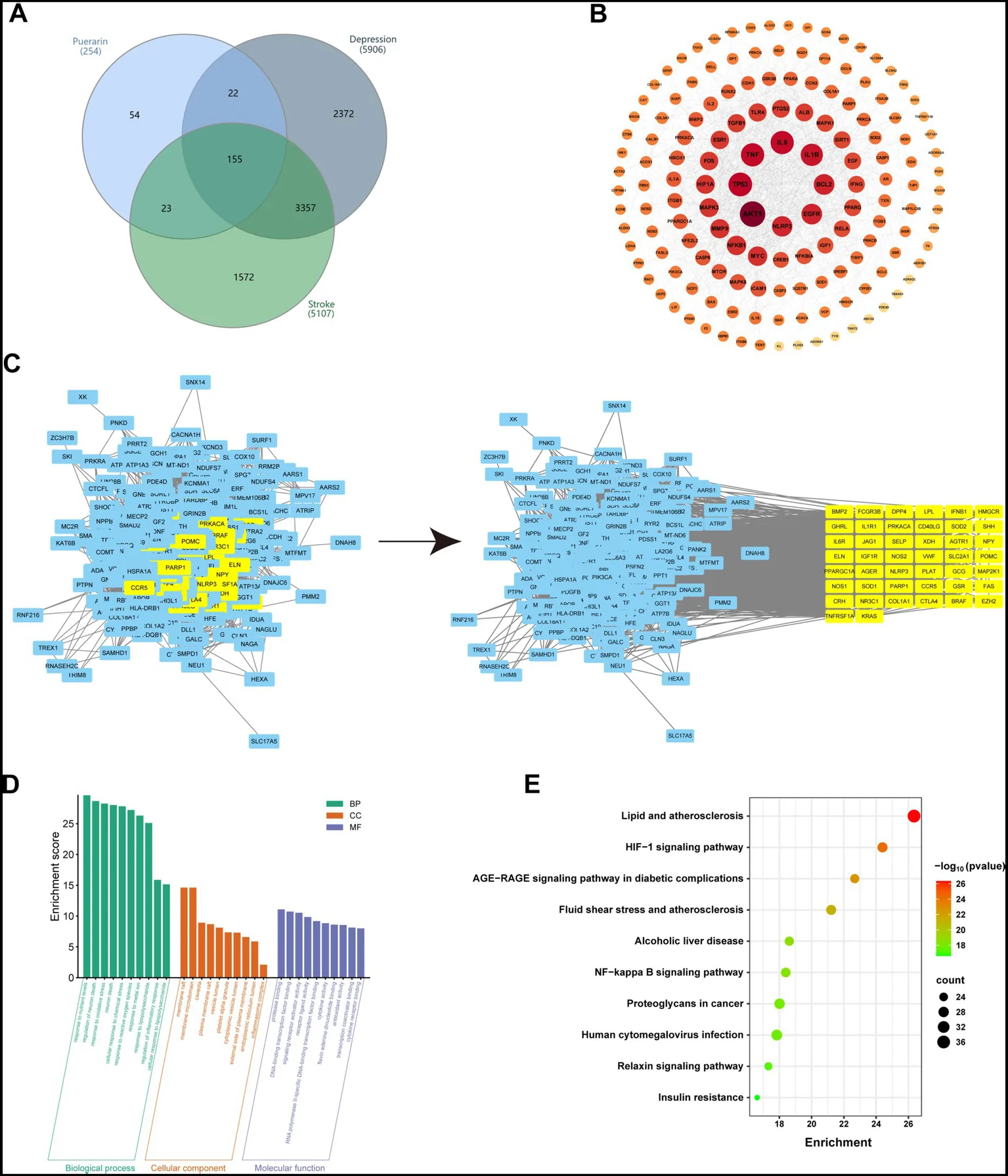
Network pharmacology analysis indicates that puerarin (PU) may modulate shared inflammatory and vascular-stress networks implicated in depression and ischemic stroke. **(A)** Venn diagram showing the predicted targets of puerarin and disease-associated targets of depression and ischemic stroke: The overlap represents common targets. **(B)** Protein–protein interaction (PPI) network of the intersecting targets: Node size and color are proportional to node degree. **(C)** Hub-target screening based on PPI topology: Hub nodes are highlighted in yellow, and the top-ranked hub targets are summarized in the table (right). **(D)** Gene Ontology (GO) enrichment of the intersecting targets across Biological Process (BP), Cellular Component (CC), and Molecular Function (MF). **(E)** Bubble plot of the top enriched KEGG pathways: Bubble size indicates the number of targets, color denotes −log10(*p* value), and the *x*-axis represents enrichment.

Network pharmacology analysis identified 155 shared targets among PU, depression, and ischemic stroke (Figure 4A). A PPI network constructed from these common targets revealed a prominent inflammatory core; TNF, IL-6, IL-1β, and NFKB1/RELA, together with NLRP3, were prioritized as highly connected hub nodes, alongside signaling regulators such as AKT1, EGFR, MYC, and TP53 (Figure 4B). Subsequent topological filtering further refined a set of key candidates, including NLRP3 and inflammation- and vascular stress-associated genes (e.g., IL1R1, IL6R, AGER, PPARGC1A, NOS1/NOS2, PARP1, CCR5, TNFRSF1A, PLAT, and VWF) (Figure 4C). GO enrichment indicated that these targets are mainly involved in oxidative/chemical stress responses, regulation of inflammatory responses, and neuron death, and are localized to structures relevant to inflammatory signaling assembly, including membrane rafts/microdomains and the inflammasome complex (Figure 4D). KEGG pathway enrichment further showed significant enrichment in lipid and atherosclerosis, HIF-1 signaling, AGE-RAGE signaling in diabetic complications, fluid shear stress and atherosclerosis, and the NF-kB signaling pathway, with additional enrichment in pathways linked to systemic stress and metabolism such as insulin resistance (Figure 4E). Collectively, these systems-level results support NLRP3 as a plausible mechanistic node through which PU may modulate inflammatory and vascular-stress networks in PSD, providing a rationale for subsequent validation focusing on the NLRP3/CASPASE-1 inflammasome axis and related metabolic disturbances.

### 3.4 PU suppressed the NLRP3/CASPASE-1 inflammasome and the generation of inflammatory cytokines in the hippocampus of PSD mice

**Figure 5:**
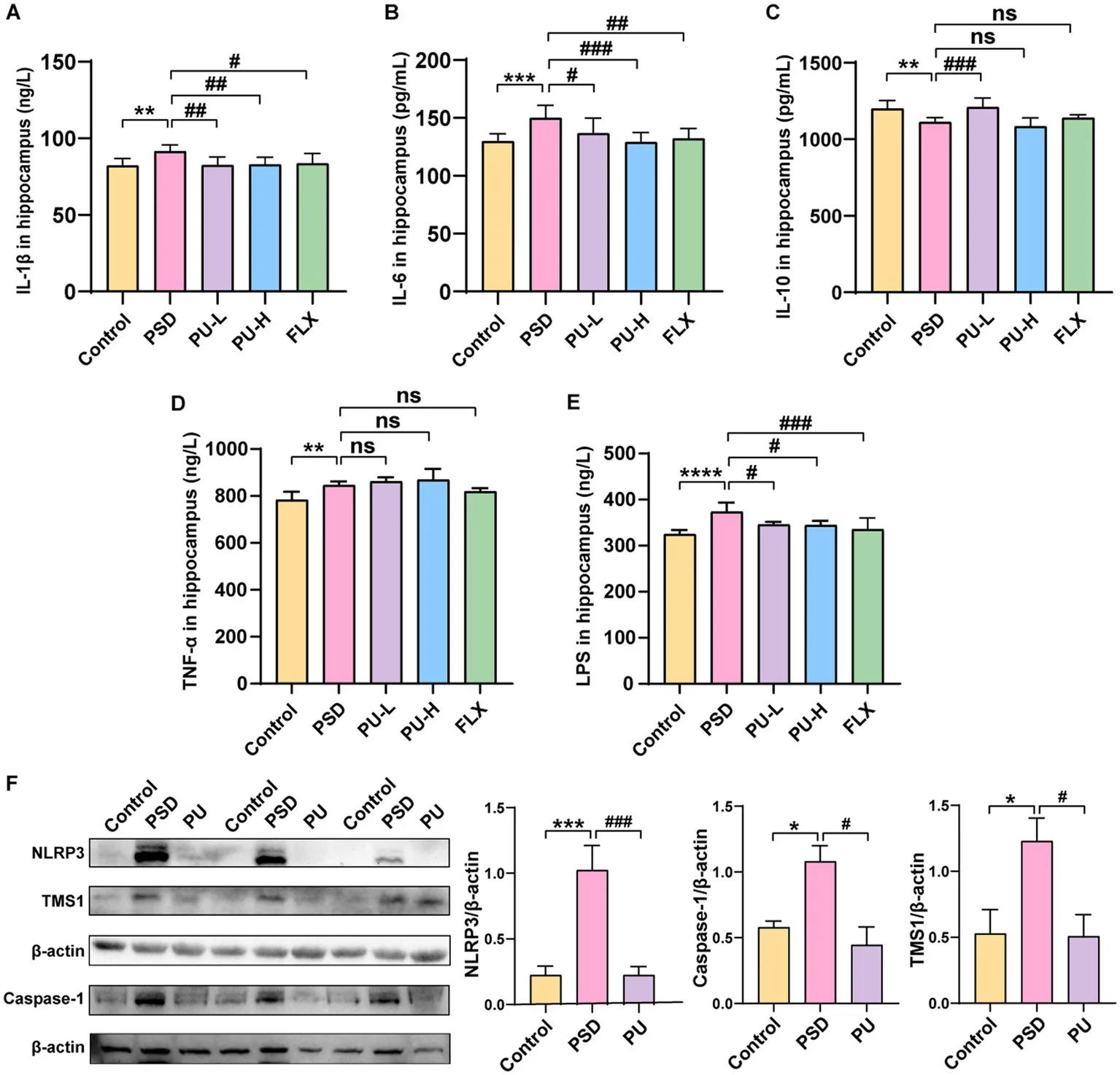
PU regulated the NLRP3/caspase-1 inflammasome in the hippocampus of PSD mice. **(A–E)** Levels of inflammatory factors, including IL-1β, IL-6, IL-10, LPS, and TNF-α, in the hippocampus (*n* = 8 per group). **(F)** Levels of the NLRP3/caspase-1 inflammasome-associated proteins in the hippocampus (*n* = 3 per group). Data are presented as the mean ± SEM. ^*^p < 0.05, ^**^p < 0.01, ^***^p < 0.001, ^****^p < 0.0001.

To further explore the changes in the hippocampus of PSD mice, the production of anti-inflammatory cytokine IL-10, as well as the pro-inflammatory cytokines IL-6, IL-1β, and TNF-*α*, together with LPS, in the hippocampus of mice, was detected by ELISA. The results suggested that, compared with the Control group, the expression of IL-1β, IL-6, TNF-α, and LPS in the hippocampus of PSD mice was significantly increased (*p* < 0.01; *p* < 0.001; *p* < 0.01; *p* < 0.0001), whereas the IL-10 level was significantly decreased (*p* < 0.01) (Figures 5A–C). The hippocampal IL-1β levels in the PU-L and PU-H groups were significantly decreased (*p* < 0.01; *p* < 0.01) (Figure 5A), whereas the hippocampal IL-6 level in the PU-L group was significantly reduced compared with that in the PSD group (*p* < 0.05) (Figure 5B). In addition, the hippocampal IL-10 level in the PU-L group was significantly higher than that in the PSD group (*p* < 0.001) (Figure 5C). Although hippocampal TNF-α levels failed to show significant improvement in all groups (Figure 5D), the hippocampal LPS levels in the PU-L, PU-H, and FLX groups were significantly reduced compared with those in the PSD group (*p* < 0.05; *p* < 0.05; *p* < 0.001) (Figure 5E). These results indicate that PU could significantly inhibit the expression of pro-inflammatory cytokines IL-6 and IL-1β, as well as LPS, in PSD mice, while promoting the generation of the anti-inflammatory cytokine IL-10, effectively alleviating hippocampal and systemic inflammation in PSD mice.

Furthermore, based on the previous network pharmacology results, the expression of hippocampal proteins including NLRP3, TMS-1, and CASPASE-1 was detected by WB. The results suggested that the expression of NLRP3, TMS-1, and CASPASE-1 proteins in the hippocampus of the PSD group was significantly increased compared with that of the Control group (*p* < 0.001; *p* < 0.05; *p* < 0.05). In contrast, the expression of NLRP3, ASC, and CASPASE-1 in the hippocampus of the PU-L group was significantly decreased compared with that in the PSD group (*p* < 0.001; *p* < 0.05; *p* < 0.05) (Figure 5F).

### 3.5 PU reconstructed the gut microbiota of PSD mice

**Figure 6:**
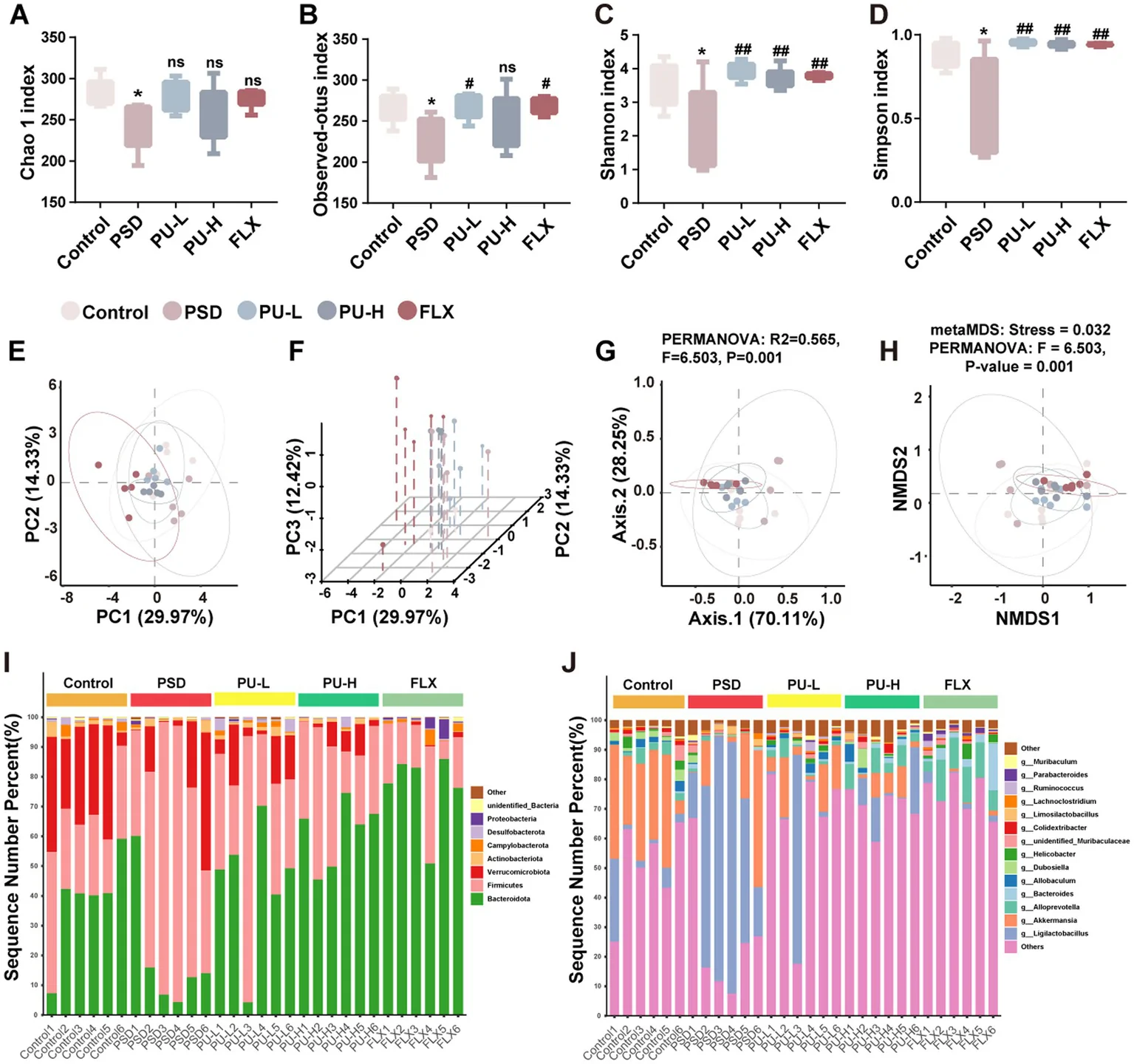
PU modulated the diversity and composition of the gut microbiota in PSD mice. **(A–D)** Box plot of the alpha-diversity indices, including Chao1, Observed_OTUs, Shannon, and Simpson. **(E,F)** Beta-diversity analyses of the gut microbiota. **(G,H)** PERMANOVA analysis showing significant differences among the groups. **(I)** Gut microbiota composition at the phylum level (bar plot). **(J)** Gut microbiota composition at the phylum level (Circos plot). **(K)** Gut microbiota composition at the genus level (bar plot). **(L)** Heat map of the gut microbiota at the genus level.

The fecal microbiota of mice was sequenced and analyzed by 16S rRNA amplicon sequencing. There were 3,024,774 raw sequencing reads obtained from 30 mouse fecal samples (*n* = 6 per group), and 2,827,721 effective tags were obtained by filtering and splicing. The changes in microbial diversity in each group were evaluated according to the alpha-diversity analysis. The Chao1 index showed that the microflora abundance in the PSD group was significantly lower than that in the Control group (*p* < 0.05), while there was no discernible difference among the PU-L, PU-H, FLX, and Control groups (Figure 6A). The Observed_OTUs index showed that, relative to the Control group, the number of observed taxa in the PSD mice was significantly reduced (*p* < 0.05), and this reduction in the PU-L and FLX groups was significantly reversed (*p* < 0.05; *p* < 0.05) (Figure 6B). The Shannon index suggested that, compared with the Control group, the diversity and evenness of the gut microbiota in the PSD group were significantly reduced (*p* < 0.05), while those of the PU-L, PU-H, and FLX groups were significantly improved (*p* < 0.01; *p* < 0.01; *p* < 0.01) (Figure 6C). The Simpson index showed similar results (Figure 6D). The above results confirmed that the abundance, diversity, and evenness of the gut microbiota in the PSD group were significantly lower than those in the Control group, while these changes could be significantly reversed by PU or FLX intervention. Combined with the beta-diversity analysis, the PCoA analysis suggested that the gut microbiota in the PSD and Control groups were evidently different, whereas those of the PU-L, PU-H, FLX, and Control groups were more similar (Figures 6E,F). PCA and NMDS analyses showed similar results (Figures 6G,H).

**Figure 7:**
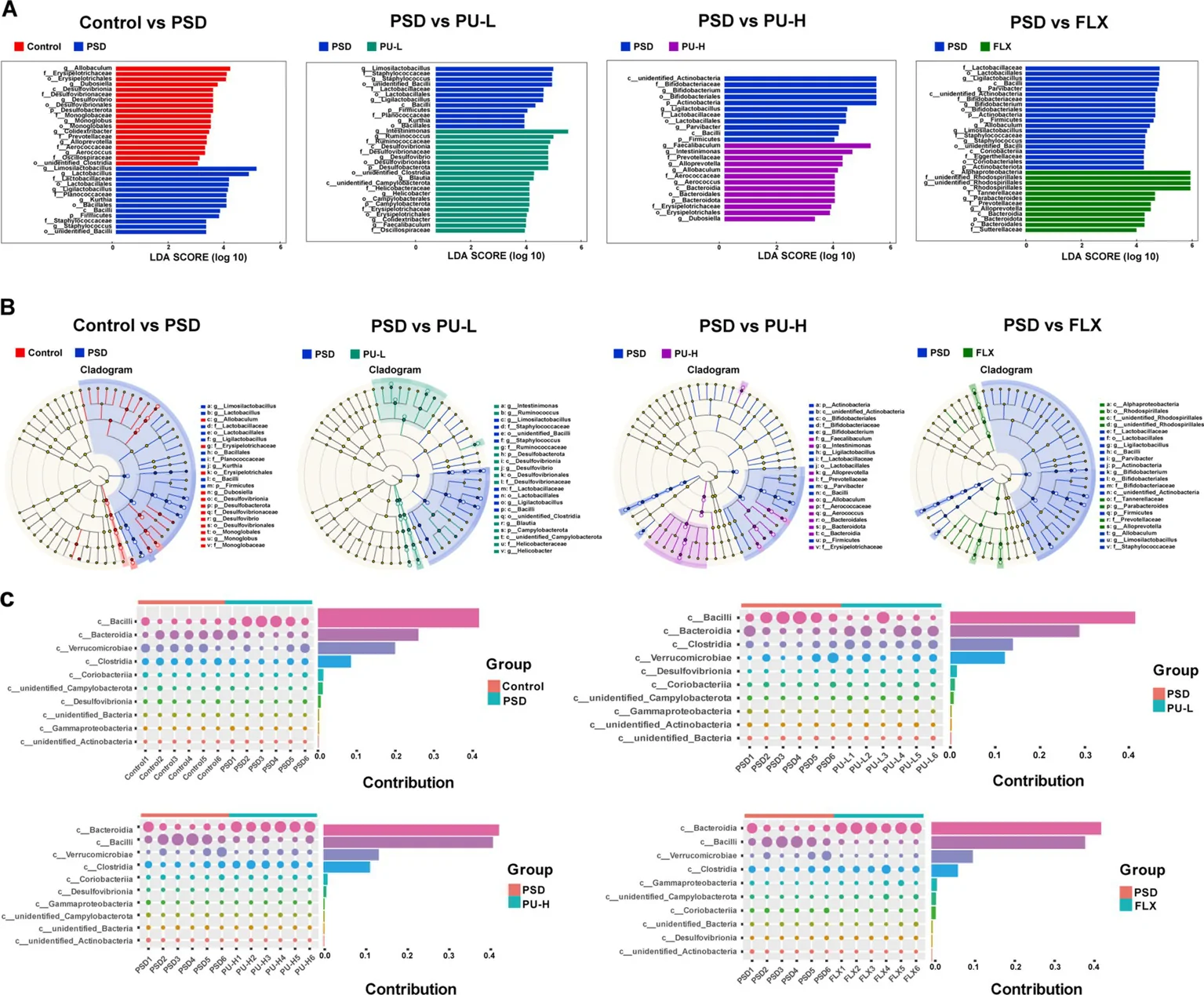
Gut microbiota changes in different groups. **(A)** Cladograms generated by LEfSe indicate differences in the bacterial taxa among the Control, PSD, PU-L, PU-H, and FLX groups. **(B)** Taxonomic cladogram from LEfSe analysis. **(C)** The SIMPER algorithm was used to generate a plot of the top 10 contributions.

A total of 10 phyla and 56 genera showed significant inter-group differences (*p* < 0.05). A bar plot was drawn according to the species abundance table (top 10 phyla and genera) (Figures 6I–L). By combining linear discriminant analysis (LDA) effect size (LEfSe), significant inter-group differences in the intestinal microbiota were identified, and the enrichment of differentially abundant microbiota was determined. The LDA score distribution histogram showed that 48 biomarkers differed significantly among the groups (*p* < 0.05, LDA > 4.0) (Figure 7A) and 19 biomarkers were identified by the cladogram (Figure 7B). In addition, the contribution of significantly altered microbiota to the differences among the groups was quantified by similarity percentage (SIMPER) analysis (Figure 7C).

In summary, compared with the Control group, the PSD group showed marked dysbiosis of the intestinal microbiota: the abundances of *Ligilactobacillus*, *Limosilactobacillus*, and *Firmicutes* were significantly increased, whereas those of *Colidextribacterium*, *Aerococcus*, *Monoglobus*, *Allobaculum*, and *Alloprevotella* were significantly reduced. PU reconstructed the intestinal microbiota of PSD mice, significantly reducing the abundances of *Firmicutes*, *Ligilactobacillus*, and *Limosilactobacillus,* while increasing the abundances of *Campylobacterota*, *Colidextribacterium*, *Allobaculum*, *Monoglobus*, *Alloprevotella*, *Faecalibaculum*, and *Aerococcus*, thereby restoring the intestinal microbiota composition.

### 3.6 PU modulated the metabolism of gut microbiota

**Figure 8:**
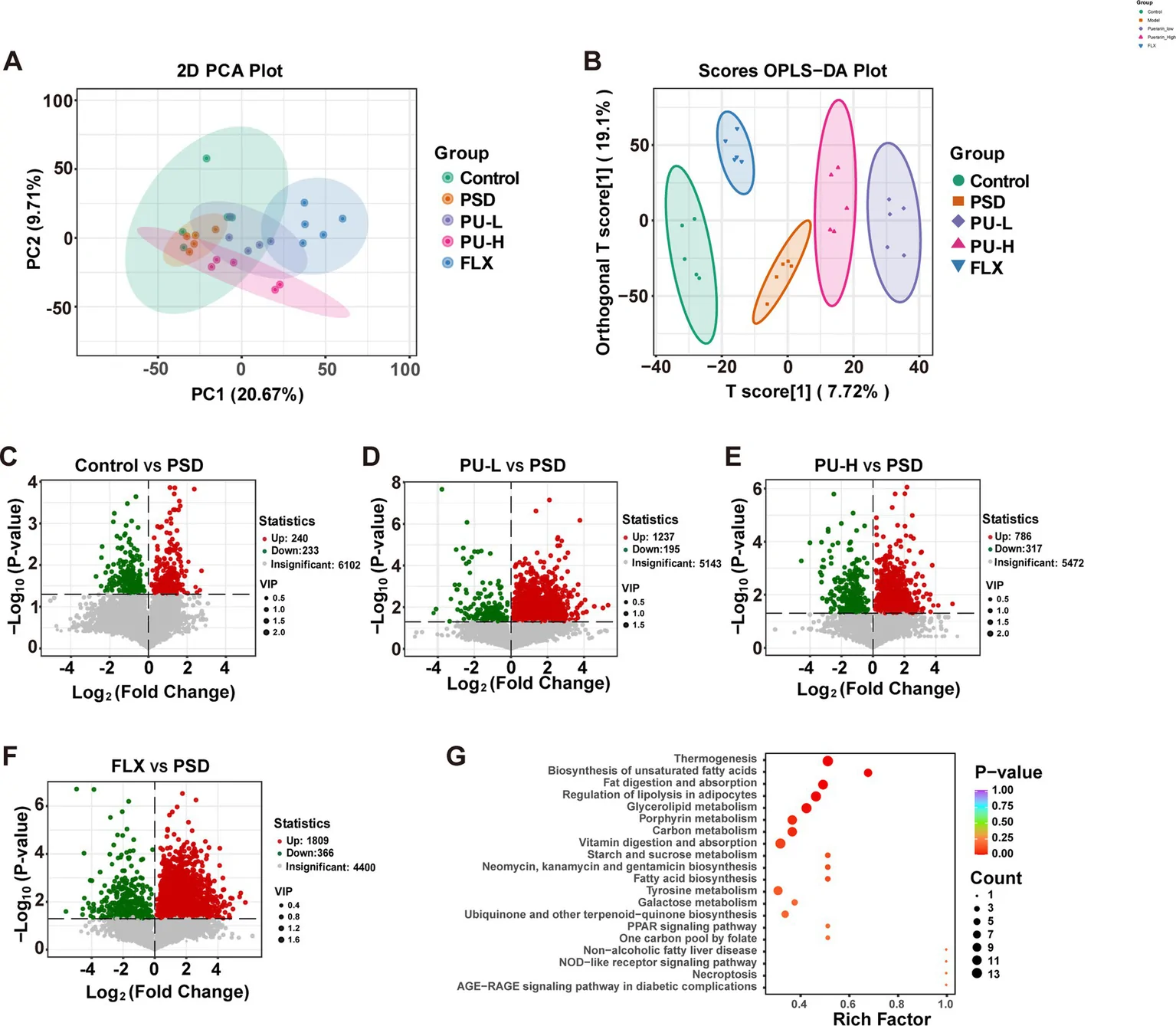
PU modulated the gut metabolites of PSD mice. **(A)** Principal component analysis (PCA) score plots showing discrimination of the fecal metabolome among the Control, PSD, PU-L, PU-H, and FLX groups. **(B)** OPLS-DA score plot. The horizontal axis shows the separation between groups, whereas the vertical axis shows the variation within groups. **(C–F)** Volcano plots of fecal metabolomics comparing the Control and PSD groups **(C)**, PU-L and PSD groups **(D)**, PU-H and PSD groups **(E)**, and FLX and PSD groups **(F)**. **(G)** Bubble plot of KEGG differential metabolite enrichment.

To further investigate the metabolic differences in the gut microbiota of PSD mice and the regulatory effects of PU on intestinal metabolites, untargeted metabolomics was performed to detect metabolites in mouse fecal samples, obtain quantitative information, identify metabolites with significant inter-group differences, and explore their association with biological processes. A total of 25 fecal samples (*n* = 5) were analyzed, and 7,689 metabolites were detected. Principal component analysis (PCA) (Figure 8A) and orthogonal partial least squares discriminant analysis (OPLS-DA) (Figure 8B) showed an obvious trend of separation in metabolite profiles among the groups; however, the differences were not statistically significant. According to the OPLS-DA model, the variable importance in projection (VIP; VIP > 1) was used for the preliminary screening of differential metabolites. Combined with univariate analysis, including hypothesis testing (*p* < 0.05) and fold-change (FC) analysis, differential metabolites were further screened. The results showed that, compared with the Control group, 240 metabolites were significantly decreased and 233 were significantly increased in the PSD group. Compared with the PSD group, 195 metabolites were significantly decreased and 1,237 were significantly increased in the PU-L group. Similarly, 317 metabolites were significantly decreased and 786 were significantly increased in the PU-H group, whereas 366 metabolites were significantly decreased and 1,809 were significantly increased in the FLX group (Figures 8C–F). Metabolite enrichment analysis based on the KEGG database indicated significant differences among the groups in pathways including glycerolipid metabolism, biosynthesis of unsaturated fatty acids, thermogenesis, regulation of lipolysis in adipocytes, and fat digestion and absorption (Figure 8G).

### 3.7 Relevance analysis between biochemical indexes and gut microbiota

To further explore the associations between biochemical indexes and the gut microbiota of mice, this study performed Pearson correlation analysis between the gut microbiota (top 10 phyla and genera) and depressive-like behaviors (SPT, OFT, FST, and TST), intestinal metabolites, and the inflammatory cytokines IL-10, IL-6, and IL-1β in the hippocampus and serum.

**Figure 9:**
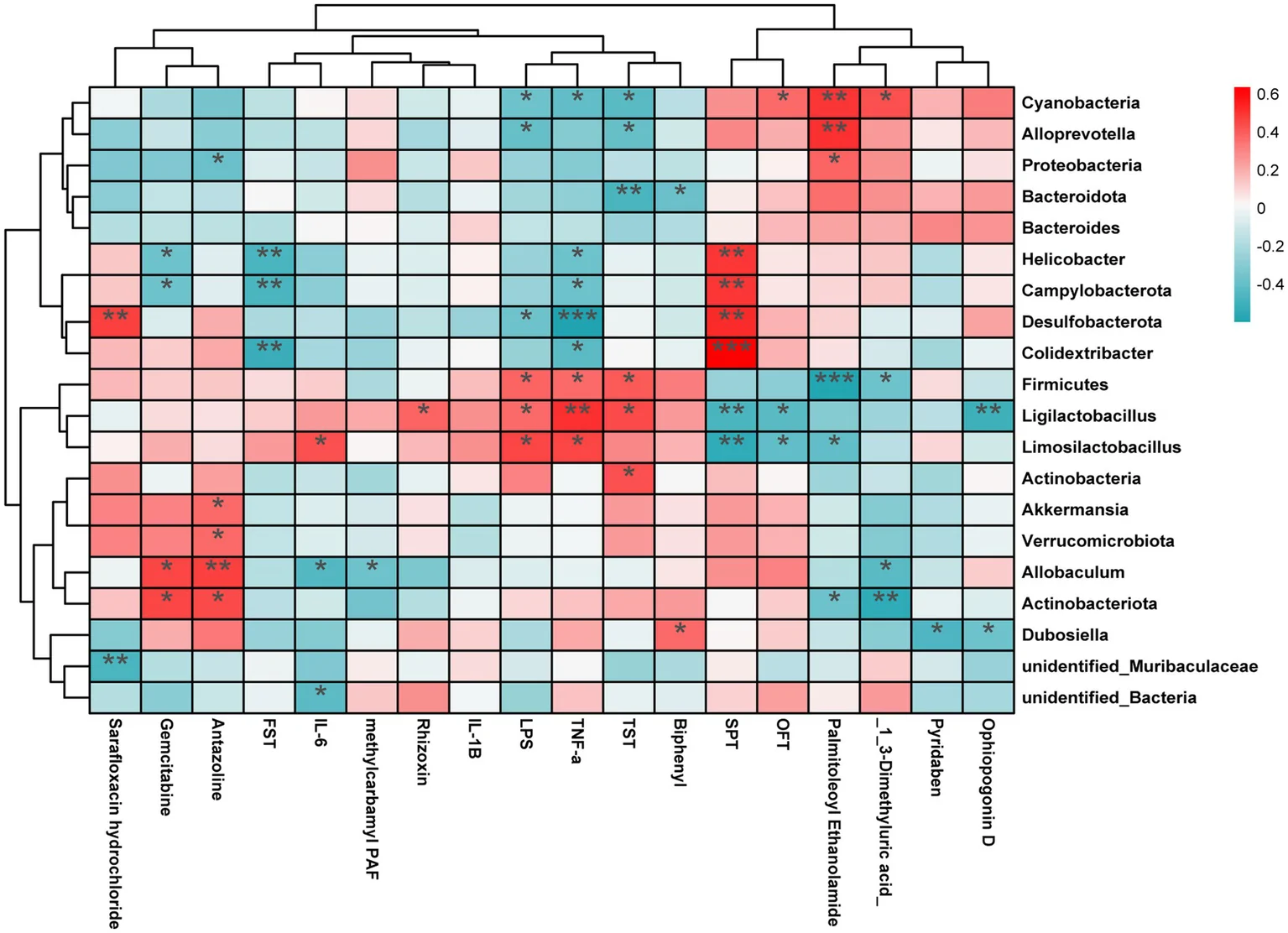
Pearson correlation analysis between the intestinal microbiota and biochemical parameters. Pearson correlation analysis between the intestinal microbiota and behavioral measures (OFT, SPT, FST, and TST), gut microbial metabolites, and inflammatory cytokines in the hippocampus and serum in mice with significant inter-group differences. *, *p* < 0.05; **, *p* < 0.01; ***, *p* < 0.001.

The results showed that *Firmicutes* was positively correlated with TST (*p* < 0.05) and negatively correlated with 1,3-dimethyluric acid and palmitoleoylethanolamide (*p* < 0.05; *p* < 0.001). *Limosilactobacillus* was positively correlated with hippocampal IL-6 and serum IL-1β and IL-6 (*p* < 0.05; *p* < 0.05; *p* < 0.01) and negatively correlated with SPT, OFT, and palmitoleoylethanolamide (*p* < 0.01; *p* < 0.05; *p* < 0.05). *Ligilactobacillus* was positively correlated with TST immobility time, Rhizoxin, and serum IL-6 (*p* < 0.05; *p* < 0.05; *p* < 0.05; *p* < 0.01) and negatively correlated with SPT, OFT, and ophiopogonin D (*p* < 0.01; *p* < 0.05; *p* < 0.01). *Campylobacterota* was positively correlated with SPT (*p* < 0.01) and negatively correlated with Gemcitabine and FST (*p* < 0.05; *p* < 0.01). *Allobaculum* was positively correlated with Gemcitabine and Antazoline (*p* < 0.05; *p* < 0.01) and negatively correlated with serum IL-1β, hippocampal IL-6, methylcarbamyl platelet-activating factor, and 1,3-dimethyluric acid (*p* < 0.05; *p* < 0.01; *p* < 0.05; *p* < 0.05). *Alloprevotella* was positively correlated with palmitoleoylethanolamide (*p* < 0.01) and negatively correlated with TST immobility time (*p* < 0.05; *p* < 0.05) (Figure 9).

### 3.8 PU-FMT effectively improved the depressive-like behaviors of PSD mice

Previous results showed that PU could ameliorate the depressive-like behaviors of PSD mice and regulate the intestinal microbiota and their metabolites. To further verify that regulation of the intestinal microbiota is a key mechanism by which PU improves the depressive-like behaviors of PSD mice, we used mice in the Control group as recipients and transplanted fecal microbiota from PSD mice into them. In addition, PSD mice were used as recipients and transplanted with fecal microbiota from PU-treated PSD mice to observe the behavioral changes among the groups (Figure 2A). The results of the OFT indicated that, compared with the Control group, the PSD-FMT group showed a significant reduction in the frequency of entering the central region and the time spent in the central region (*p* < 0.01; *p* < 0.001) (Figures 2B–E). Compared with the PSD group, the frequency of entering the central region and the time spent in the central region were significantly increased in the PU-FMT group (*p* < 0.05; *p* < 0.05) (Figures 2B–E). The results of the SPT indicated that, compared with the Control group, the sucrose preference of the PSD and PSD-FMT groups was significantly decreased (*p* < 0.001; *p* < 0.001), whereas the sucrose preference of the PU-FMT group was significantly higher than that of the PSD group (*p* < 0.05) (Figure 2F). The results of TST indicated that the immobility time of the PSD-FMT and PSD groups was significantly prolonged compared with that of the Control group (*p* < 0.05; *p* < 0.05), whereas the immobility time of the PU-FMT group was significantly shortened compared with that of the PSD group (*p* < 0.01) (Figure 2G). The results of the FST showed that the immobility time of the PSD-FMT and PSD groups was significantly prolonged compared with that of the Control group (*p* < 0.05; *p* < 0.05), whereas the immobility time of the PU-FMT group was significantly shortened compared with that of the PSD group (*p* < 0.05) (Figure 2H). In conclusion, according to the behavioral test results, compared with the Control group, the PSD and PSD-FMT groups exhibited significant depressive-like behaviors, whereas the PU-FMT group showed significant improvement, suggesting that the intestinal microbiota is a key mediator of the antidepressant effects of PU in PSD mice.

## 4 Discussion

**Figure 10:**
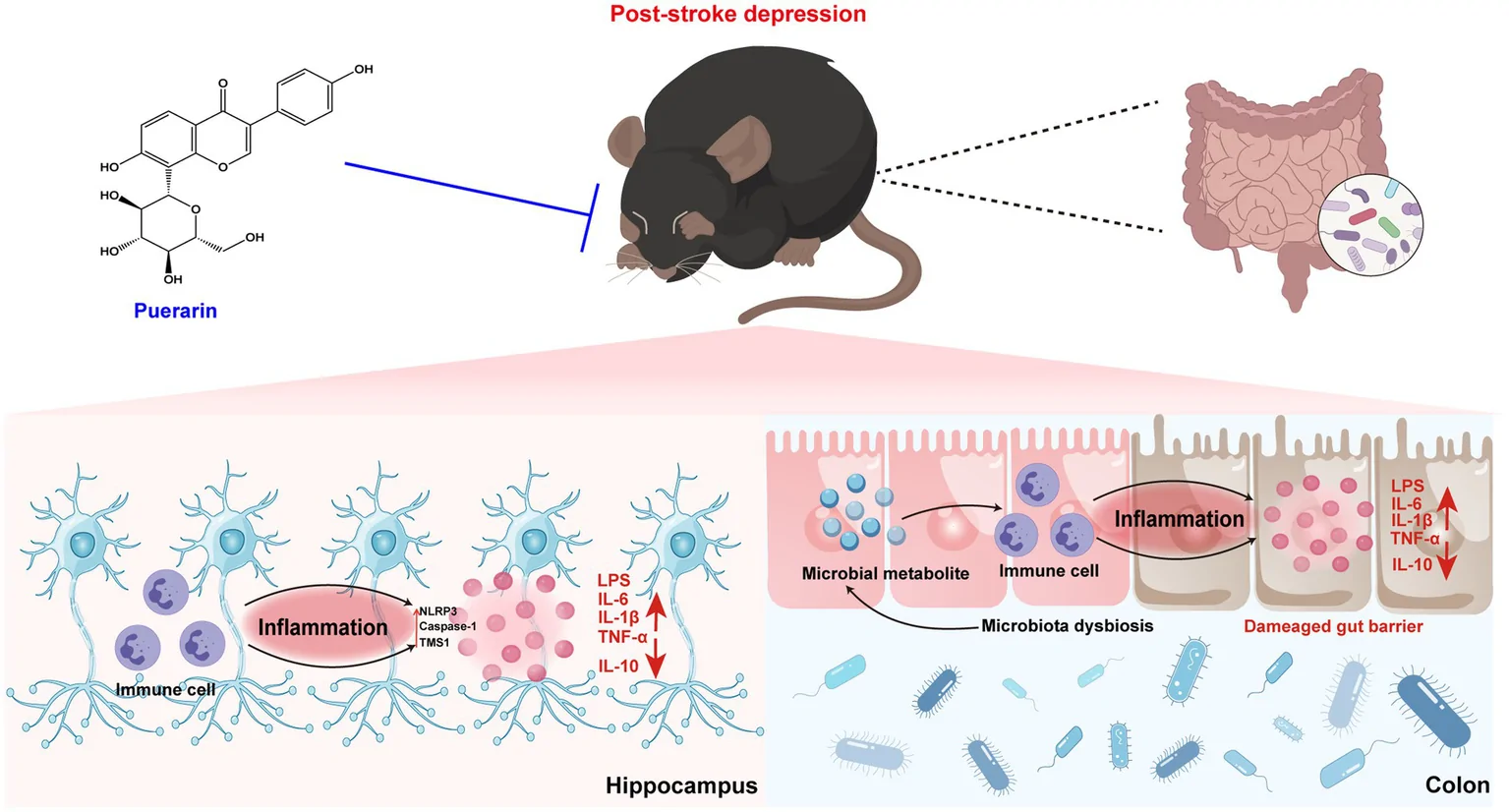
Puerarin improves depressive-like behaviors in post-stroke depression mice via modulating the gut microbiota and microbial metabolism.

The present results suggest that PU could effectively alleviate the depressive-like behaviors of PSD mice, repair gut microbiota dysbiosis and microbial metabolic disorders, reduce the inflammatory response in the hippocampus and colon, increase the expression of occludin and ZO-1, and suppress the NLRP3/CASPASE-1 inflammasome. PSD-FMT further demonstrated that gut microbiota disruption is a key etiological factor in the pathological progression of PSD. Meanwhile, PU-FMT demonstrated that PU could substantially alleviate the depressive-like behaviors of PSD mice, suggesting that PU exerts its antidepressant effects in PSD mice by regulating the intestinal microbiota (Figure 10).

Stroke is a cardiovascular and cerebrovascular disease with high mortality and disability rates, and PSD is a common psychological disorder in stroke patients that seriously reduces recovery and quality of life (Zhou et al., 2024). Although PSD has received widespread attention in the medical field, its specific pathogenesis remains unclear, which seriously limits the development of improved clinical treatments. Studies have shown that PU therapy can significantly improve the symptoms of stroke patients and promote the recovery of neurological function (Liu X. et al., 2023b); however, studies investigating its potential to improve the prognosis of PSD remain limited. This study aimed to evaluate the effects of PU on the depressive-like behaviors of PSD mice. A comprehensive battery of behavioral tests was used to assess the effects of low and high doses of PU on PSD mice. The results indicated that, compared with the Control group, the PSD group showed significant depressive-like behaviors, whereas the PU-L, PU-H, and FLX groups exhibited varying degrees of improvement, with the PU-L group showing the greatest improvement. These findings demonstrate that PU is an effective therapeutic agent for alleviating depressive-like behaviors in PSD mice.

Previous studies have found a link between changes in the gut microbiota and the pathological progression of PSD. Disturbances in the gut microbiota affect the nervous system through neural, endocrine, and metabolic pathways, thereby leading to the development of depressive-like behaviors (Shi et al., 2024). At the same time, PU could relieve colonic inflammation and restore intestinal function by restoring the structure of the gut microbiota (Zou et al., 2023). Moreover, it could effectively ameliorate the depressive-like behaviors of CUMS mice by reshaping the gut microbiota (Song et al., 2021). In addition, numerous experimental studies have demonstrated that Chinese herbal formulas and their monomeric components can remodel the gut microbiota and suppress excessive activation of the NLRP3 inflammasome and subsequent inflammatory responses, thereby alleviating depressive-like behaviors in PSD mice. For example, our research group has demonstrated that the classic herbal prescription Xiaoyaosan markedly relieves depressive-like behaviors in PSD mice by modulating the gut microbiota and inhibiting the NLRP3/P2X7R signaling pathway (Chen Y. Y. et al., 2025). Morinda officinalis oligosaccharides mitigate hippocampal neuroinflammation and improve behavioral performance in PSD rats via downregulation of NLRP3 expression in hippocampal microglia (Li et al., 2021). Multiple natural monomer compounds, including astragaloside IV, curcumin, and ginsenoside Rd., exert similar regulatory effects to ameliorate PSD manifestations (Che et al., 2026). However, existing research on the role of the gut microbiota in PSD is still at an early stage, and there is a lack of evidence as to whether PU regulates gut microbial metabolism to treat PSD.

This study characterized the intestinal microbiota of mice by 16S rRNA amplicon sequencing. The alpha-diversity and beta-diversity analyses showed that, compared with the Control group, the abundance, diversity, and evenness of the gut microbiota were significantly reduced in PSD mice, and these changes were reversed by PU or FLX treatment. These findings suggest that PSD mice are characterized by gut microbiota dysbiosis and that PU could effectively restore the balance of the intestinal microbiota. To further verify that gut microbiota dysbiosis is a key factor in the pathogenesis of PSD, FMT was performed using fecal microbiota from PSD mice transplanted into Control mice, resulting in obvious depressive-like behaviors. These findings suggest that gut microbiota dysbiosis contributes to the progression of PSD. Meanwhile, FMT was performed in PSD mice using fecal microbiota from PU-treated mice, and a significant improvement in depressive-like behaviors was observed. Given that PU could treat PSD by restoring the balance of the intestinal microbiota, this study further identified intestinal microbiota with significant inter-group differences at the phylum and genus levels. The results indicated that 10 phyla and 56 genera differed significantly among the groups. Compared with the Control group, PSD mice exhibited obvious gut microbiota dysbiosis, with significantly increased abundances of *Ligilactobacillus, Limosilactobacillus,* and *Firmicutes*, whereas the abundances of *Colidextribacterium*, *Aerococcus*, *Monoglobus*, *Allobaculum*, and *Alloprevotella* were significantly reduced. PU restored the intestinal microbiota of PSD mice by significantly reducing the abundances of *Firmicutes*, *Ligilactobacillus*, and *Limosilactobacillus*, while significantly increasing the abundances of *Campylobacterota*, *Colidextribacterium*, *Allobaculum*, *Monoglobus*, *Alloprevotella*, *Faecalibaculum*, and *Aerococcus*, thereby restoring the intestinal microbiota composition. Recent studies have confirmed that *Firmicutes,* one of the major bacterial phyla of the healthy human gut, shows significant alterations in abundance in PSD (Sun et al., 2022; Jiang et al., 2021). *Firmicutes* can participate in lipid metabolism by regulating the short-chain fatty acids, thereby influencing the development of PSD (Jiang et al., 2023). *Limosilactobacillus* and *Ligilactobacillus* are widely recognized as probiotic bacteria that resist pathogens, modulate immunity, and maintain intestinal mucosal integrity (Ozen et al., 2023). However, this property of probiotics does not always mean that they have the ability to promote health. Excessive proliferation of probiotics that disrupts the balance of the intestinal microbiota may induce allergic reactions, metabolic disorders, and gastrointestinal dysfunction (Doron and Snydman, 2015). Notably, this study showed that the abundances of *Limosilactobacillus* and *Ligilactobacillus* were significantly increased in the intestines of PSD mice. *Colidextribacter* has been shown to enhance the intestinal barrier and inhibit colonic inflammation (Liu X. et al., 2022). *Allobaculum* contributes to the improvement of vascular endothelial dysfunction in hypertension and also helps maintain intestinal homeostasis and alleviate colonic inflammation (Liu T. et al., 2023a; Mo et al., 2022). *Monoglobus* and *Alloprevotella* are conditionally pathogenic bacteria that can be found in the normal human gut and participate in intestinal metabolism to some extent (Kong C. et al., 2019). Meanwhile, in this study, we also analyzed fecal metabolite alterations in the different groups by untargeted metabolomics and identified metabolites with significant inter-group differences, including pyridaben, gemcitabine, ophiopogonin D, palmitoleoylethanolamide, 1,3-dimethyluric acid, and biphenyl. KEGG pathway analysis revealed significant inter-group differences in the regulation of lipolysis in adipocytes, fat digestion and absorption, thermogenesis, biosynthesis of unsaturated fatty acids, and glycerolipid metabolism.

A healthy, intact intestinal mucosal barrier is vital for maintaining the stability of the intestinal environment. Pathological damage to the intestinal mucosa leads to the diffusion of inflammatory factors from the intestine into the circulation, which, in turn, induces systemic inflammatory responses. Therefore, we examined the intestinal mucosal structure in each group of mice by electron microscopy and HE staining. In addition, we detected the expression levels of tight-junctional proteins occludin and ZO-1 in the colonic mucosa by WB. The expression of occludin and ZO-1 in PSD mice was significantly decreased, whereas PU treatment significantly increased the expression of occludin and ZO-1 in PSD mice. The results confirmed that the intestinal mucosa of PSD mice showed a more severe inflammatory response and other pathological damage than that of the Control group. After PU treatment, the integrity of the intestinal mucosa was effectively restored, and the inflammatory response was controlled.

Previous research has confirmed that the gut microbiota plays a critical role in the immune system and that there is a potential correlation between inflammasome activation and gut microbiota dysbiosis. The NLRP3/CASPASE-1 inflammasome is a classic inflammasome signaling pathway involved in both peripheral and central inflammatory responses. Numerous studies have suggested that activation of the NLRP3/CASPASE-1 inflammasome is a key pathological factor in the onset of PSD (Cai et al., 2024). Network pharmacology is an important method for identifying therapeutic targets. Interestingly, we found that the NLRP3/CASPASE-1 inflammasome is a potential therapeutic target of PU in PSD. This is consistent with the published consensus in this field that the specific gut microbiota and corresponding metabolic signatures shaped by drug intervention can be stably transferred to recipient mice via FMT, thereby regulating the activation of the central NLRP3 inflammasome (Chen Y. et al., 2025; Han et al., 2025). Consistently, this research found that, compared with the Control group, the expression of NLRP3, ASC, and CASPASE-1 in the hippocampus of PSD mice was significantly increased. In contrast, PU treatment significantly reduced the expression of these proteins, suggesting that the activation of the NLRP3/CASPASE-1 inflammasome in the hippocampus was effectively suppressed and hippocampal neuroinflammation was alleviated. We also measured the levels of inflammatory cytokines in the serum and hippocampus, including IL-10, IL-6, IL-1β, LPS, and TNF-*α*. The results showed that PU increased IL-10 levels and decreased IL-6, IL-1β, LPS, and TNF-α, effectively alleviating the inflammatory response in PSD. IL-1β is an important indicator of NLRP3/CASPASE-1 inflammasome activation. Recent studies have indicated that the pro-inflammatory cytokines IL-6 and IL-1β play important roles in the progression of PSD. These inflammatory factors may contribute to the progression of PSD through multiple pathways, including regulation of the hypothalamic–pituitary–adrenal (HPA) axis, alteration of neurotransmitters and neurotrophic factors, and promotion of gut microbiota dysbiosis (Zhang et al., 2023). LPS serves as a canonical upstream agonist of the NLRP3 inflammasome. In the ischemic stroke models, cerebral ischemia-induced stress disrupts the integrity of the intestinal barrier, resulting in the translocation of substantial amounts of gut microbiota-derived LPS into the peripheral circulation. Subsequently, circulating LPS penetrates the impaired blood–brain barrier and reaches emotion- and cognition-related brain regions such as the hippocampus, thereby further activating the NLRP3 inflammasome pathway in microglia, induces persistent neuroinflammation, and ultimately participates in the occurrence and progression of emotional disorders (Ye et al., 2025; Zhou et al., 2026). Although this study showed that PU had no significant regulatory effect on hippocampal TNF-α levels, cumulative evidence from meta-analyses has revealed that stroke patients with acute-phase depression exhibit substantially higher peripheral serum TNF-α levels than stroke patients without depressive manifestations, supporting TNF-α as a potential biomarker for predicting PSD in the acute stroke stage (Chen et al., 2020). IL-10 is an important anti-inflammatory cytokine that can alleviate depressive-like behaviors and cognitive dysfunction in PSD rats by inhibiting inflammatory responses and oxidative stress (Bai et al., 2024). In addition, several other inflammatory cytokines are potentially implicated in the initiation and progression of PSD. For instance, IL-17 disrupts blood–brain barrier permeability, facilitates the infiltration of peripheral inflammatory factors into the brain, and exacerbates post-stroke neuronal injury and depressive-like behaviors (Zheng et al., 2024). Furthermore, serum IL-18 is markedly elevated at 1–3 months post-stroke, and its concentration is positively correlated with the severity of depressive symptoms, indicating that IL-18 could serve as a specific inflammatory biomarker for the medium-term prediction of PSD. Mechanistically, IL-18 may synergize with IL-1β to amplify central neuroinflammatory cascades, thereby persistently facilitating the onset and progression of PSD (Zhou et al., 2025). The role of these inflammatory factors in PSD, as well as the regulatory effect of PU on them, merits further investigation.

Furthermore, Pearson correlation analysis was performed to evaluate the correlations between the intestinal microbiota and depressive-like behaviors, gut microbial metabolites, and the levels of inflammatory cytokines in the serum and hippocampus. The results suggested that the intestinal microbiota was highly correlated with depressive-like behaviors in mice, including Firmicutes, *Limosilactobacillus, Ligilactobacillus, Campylobacterota, Allobaculum*, and *Alloprevotella*. These findings suggest that gut microbiota dysbiosis and metabolic disorders are critical pathological factors in the progression of PSD. Meanwhile, the FMT experiment further verified that gut microbiota dysbiosis is a major pathological factor contributing to the development of PSD and also demonstrated that PU could alleviate the depressive-like behaviors of PSD mice by restoring the balance of the gut microbiota.

Nevertheless, this study still has some limitations. First, although the FMT experiment verified the regulatory role of the gut microbiota, we did not examine changes in NLRP3 expression in recipient mice after FMT, which hinders the establishment of a complete causal evidence chain. Second, this study detected only a limited number of inflammatory indicators and lacked the assessment of numerous other key inflammatory mediators; corresponding Western blot assays for these target proteins should be supplemented in future research. Meanwhile, FMT, multi-omics correlation, and network pharmacology data provide only indirect associative and mediating evidence. Direct causal confirmation requires further validation using specific pathway inhibitors and NLRP3 knockout mice to determine whether the antidepressant effect of PU changes significantly, thereby verifying the necessity of the NLRP3 inflammasome pathway. Such validation experiments will be conducted as part of a follow-up independent project by our research group. In addition, we will purchase standard strains and conduct systematic single-strain gavage experiments in a separate follow-up project to clarify their underlying biological functions and regulatory effects on metabolites, as well as to confirm whether these core bacterial taxa mediate the antidepressant activity of PU. Third, the failure to simultaneously detect fecal metabolites in FMT recipient mice is also a notable limitation of the current experimental design. We will conduct an independent follow-up study to synchronously collect fecal samples from both FMT donors and recipients to fully verify the regulatory cascade of the gut microbiota–metabolites–NLRP3 inflammasome axis. Last but not least, sex exerts a profound influence on the pathogenesis of depression. Notably, such sex differences are even more pronounced with respect to the gut microbiota and its metabolites, and sex-based subgroup experiments should be further explored in future research.

In summary, the progression of PSD was closely associated with intestinal mucosal damage, gut microbiota dysbiosis and metabolic disorders, activation of the NLRP3/CASPASE-1 inflammasome, as well as dysregulation of gut–brain inflammatory cytokines. In the animal experiments, PU effectively alleviated the depressive-like behaviors and inflammation of PSD mice, restored intestinal barrier function, suppressed the activation of the NLRP3/CASPASE-1 inflammasome, reconstructed the balance of the gut microbiota, including reducing the abundance of pathogenic bacteria and increasing beneficial bacteria, and regulated the metabolites of the gut microbiota.

## Data Availability

The data presented in this paper are stored in NCBI SRA database under accession number PRJNA1501384: https://www.ncbi.nlm.nih.gov/sra/PRJNA1501384.

## Footnotes

1
http://www.swisstargetprediction.ch/

2
https://www.uniprot.org/

3
https://omim.org/

4
https://www.genecards.org/

5
https://bioinfogp.cnb.csic.es/

6
https://cytoscape.org/

7
https://string-db.org/

8
https://david.ncifcrf.gov/summary.jsp

## Glossary

CRS: Chronic restraint stress
EPM: Elevated plus-maze test
ELISA: Enzyme-linked immunosorbent assay
FMT: Fecal microbiota transplantation
FLX: Fluoxetine
FST: Forced swimming test
HE: Hematoxylin and eosin
IL-6: Interleukin-6
IL-1β: Interleukin-1β
MGB: Microbiota–gut–brain
NMDS: Non-metric multidimensional scaling
OFT: Open field test
PU: Puerarin
PSD: Post-stroke depression
PCA: Principal component analysis
PCoA: Principal coordinates analysis
SCFAs: Short-chain fatty acids
SEM: Standard error of the mean
SPT: Sucrose preference test
TMAO: Trimethylamine N-oxide
TCM: Traditional Chinese medicine
TST: Tail suspension test
UHPLC: Ultra-high performance liquid chromatography
WB: Western blotting

## References

[ref1] Bai Y. Sui R. Zhang L. Bai B. Zhu Y. Jiang H. (2024). Resveratrol improves cognitive function in post-stroke depression rats by repressing inflammatory reactions and oxidative stress via the Nrf2/HO-1 pathway. Neuroscience 541, 50–63. doi: 10.1016/j.neuroscience.2024.01.017 38278473

[ref2] Cai W. Wei X. F. Tao L. Shen W. D. (2025). Antidepressant-like effects of electroacupuncture by regulating NLRP3-mediated hippocampal inflammation and pyroptosis in rats with post-stroke depression. Brain Behav. 15:e70670. doi: 10.1002/brb3.70670, 40621695 PMC12230635

[ref3] Cai W. Wei X.-F. Zhang J.-R. Tao L. Li D. Zhang K. . (2024). Acupuncture ameliorates depression-like behavior of poststroke depression model rats through the regulation of gut microbiota and NLRP3 inflammasome in the colon. Neuroreport 35, 883–894. doi: 10.1097/wnr.0000000000002076, 39207304

[ref4] Castilla-Guerra L. Fernandez Moreno M. D. C. Esparrago-Llorca G. Colmenero-Camacho M. A. (2019). Pharmacological management of post-stroke depression. Expert. Rev. Neurother. 20, 157–166. doi: 10.1080/14737175.2020.1707666, 31860359

[ref5] Che L. Xie J. Xia C. Yu Q. (2026). Traditional Chinese medicine ameliorates depression via the gut-brain Axis: a review focus on NLRP3/TLR4-mediated inflammatory pathways and gut microbiota modulation. Neuropsychiatr. Dis. Treat. 22:579710. doi: 10.2147/NDT.S579710, 41858422 PMC12998633

[ref6] Chen Y. Y. Lu Y. T. Wang Y. D. Ding N. N. Huang Z. X. Hu J. B. . (2025). Xiaoyaosan improves depression-like behaviours in mice with post-stroke depression by modulating gut microbiota and microbial metabolism and regulating P2X7R/NLRP3 inflammasome. Phytomedicine 145:157078. doi: 10.1016/j.phymed.2025.157078, 40684490

[ref7] Chen Y. Pu J. Liu Y. Tian L. Chen X. Gui S. . (2020). Pro-inflammatory cytokines are associated with the development of post-stroke depression in the acute stage of stroke: a meta-analysis. Top. Stroke Rehabil. 27, 620–629. doi: 10.1080/10749357.2020.1755813, 32316861

[ref8] Chen Y. Yu L. Zhang L. Liu C. You Y. Guo H. . (2025). Gut microbiota dysbiosis exacerbates post-stroke depression via microglial NLRP3 inflammasome activation. Exp. Neurol. 395:115488. doi: 10.1016/j.expneurol.2025.115488, 41052746

[ref9] Doron S. Snydman D. R. (2015). Risk and safety of probiotics. Clin. Infect. Dis. 60, S129–S134. doi: 10.1093/cid/civ085, 25922398 PMC4490230

[ref10] Du R.-H. Tan J. Sun X.-Y. Lu M. Ding J.-H. Hu G. (2016). Fluoxetine inhibits NLRP3 inflammasome activation: implication in depression. Int. J. Neuropsychopharmacol. 19:pyw037. doi: 10.1093/ijnp/pyw037, 27207922 PMC5043644

[ref11] Fabi J. P. (2024). The connection between gut microbiota and its metabolites with neurodegenerative diseases in humans. Metab. Brain Dis. 39, 967–984. doi: 10.1007/s11011-024-01369-w 38848023

[ref12] Gao L.-N. Yan M. Zhou L. Wang J. A. Sai C. Fu Y. . (2021). Puerarin alleviates depression-like behavior induced by high-fat diet combined with chronic unpredictable mild stress via repairing TLR4-induced inflammatory damages and phospholipid metabolism disorders. Front. Pharmacol. 12:767333. doi: 10.3389/fphar.2021.767333, 34975477 PMC8714847

[ref13] Geusgens C. A. V. VAN Tilburg D. C. H. Fleischeuer B. Bruijel J. (2024). The relation between insomnia and depression in the subacute phase after stroke. Neuropsychol. Rehabil. 35, 757–773. doi: 10.1080/09602011.2024.2370072 38941450

[ref14] Gu P. Ding Y. Ruchi M. Feng J. Fan H. Fayyaz A. . (2024). Post-stroke dizziness, depression and anxiety. Neurol. Res. 46, 466–478. doi: 10.1080/01616412.2024.2328490, 38488118

[ref15] Han M. Zhou Y. Gao X. Cheng X. Deng L. Ji S. . (2025). Modulation of gut microbiota by Gardeniae fructus oil exerts TLR4/NF-κB/NLRP3 pathway-mediated antidepressant effects based on transcriptomics and fecal transplantation. Front. Pharmacol. 16:1635897. doi: 10.3389/fphar.2025.1635897, 40822487 PMC12354470

[ref16] Hao W. Gan H. Wang L. Huang J. Chen J. (2022). Polyphenols in edible herbal medicine: targeting gut-brain interactions in depression-associated neuroinflammation. Crit. Rev. Food Sci. Nutr. 63, 12207–12223. doi: 10.1080/10408398.2022.2099808, 35838146

[ref17] Hao W. Ma Q. Wang L. Yuan N. Gan H. He L. . (2024). Gut dysbiosis induces the development of depression-like behavior through abnormal synapse pruning in microglia-mediated by complement C3. Microbiome 12:34. doi: 10.1186/s40168-024-01756-6, 38378622 PMC10877840

[ref18] Hediyal T. A. Vichitra C. Anand N. Bhaskaran M. Essa S. M. Kumar P. . (2024). Protective effects of fecal microbiota transplantation against ischemic stroke and other neurological disorders: an update. Front. Immunol. 15:1324018. doi: 10.3389/fimmu.2024.1324018, 38449863 PMC10915229

[ref19] He H. Y. Guo T. Zhang P. Y. Yang L. Q. Deng Y. H. (2017). Puerarin provides a neuroprotection against transient cerebral ischemia by attenuating autophagy at the ischemic penumbra in neurons but not in astrocytes. Neurosci. Lett. 643, 45–51. doi: 10.1016/j.neulet.2017.02.009 28192195

[ref20] Hou Y. Liu Y. Liu C. Yan Z. Ma Q. Chen J. . (2020). Xiaoyaosan regulates depression-related behaviors with physical symptoms by modulating orexin a/OxR1 in the hypothalamus. Anatomical Record 303, 2144–2153. doi: 10.1002/ar.24386, 32175693

[ref21] Jeong S. Chokkalla A. K. Davis C. K. Vemuganti R. (2023). Post-stroke depression: epigenetic and epitranscriptomic modifications and their interplay with gut microbiota. Mol. Psychiatry 28, 4044–4055. doi: 10.1038/s41380-023-02099-8, 37188778 PMC10646155

[ref22] Jiang W. Chen J. Gong L. Liu F. Zhao H. Yan Z. . (2023). Microbiota-derived short-chain fatty acids may participate in post-stroke depression by regulating host's lipid metabolism. J. Psychiatr. Res. 161, 426–434. doi: 10.1016/j.jpsychires.2023.03.032, 37031497

[ref23] Jiang W. Gong L. Liu F. Ren Y. Mu J. (2021). Alteration of gut microbiome and correlated lipid metabolism in post-stroke depression. Front. Cell. Infect. Microbiol. 11:663967. doi: 10.3389/fcimb.2021.663967, 33968807 PMC8100602

[ref24] Kazemi A. Noorbala A. A. Azam K. Eskandari M. H. Djafarian K. (2019). Effect of probiotic and prebiotic vs placebo on psychological outcomes in patients with major depressive disorder: a randomized clinical trial. Clin. Nutr. 38, 522–528. doi: 10.1016/j.clnu.2018.04.010 29731182

[ref25] Kong C. Gao R. Yan X. Huang L. Qin H. (2019). Probiotics improve gut microbiota dysbiosis in obese mice fed a high-fat or high-sucrose diet. Nutrition 60, 175–184. doi: 10.1016/j.nut.2018.10.002 30611080

[ref26] Kong H. Zhang G. Cheng J. Shi R. Zhang M. Cao P. . (2019). Distribution kinetics of puerarin in rat hippocampus after acute local cerebral ischemia. J. Pharm. Biomed. Anal. 164, 196–201. doi: 10.1016/j.jpba.2018.10.038, 30390562

[ref27] Lam Ching W. Li H. J. Guo J. Yao L. Chau J. Lo S. . (2023). Acupuncture for post-stroke depression: a systematic review and network meta-analysis. BMC Psychiatry 23:314. doi: 10.1186/s12888-023-04749-1, 37143014 PMC10161596

[ref28] Le H. T. Honma K. Annaka H. Sun S. Nomura T. (2024). Effectiveness of problem-solving therapy in improving patient mental health, function, quality of life, and mortality post-stroke: a systematic review. Behav. Sci. 14:446. doi: 10.3390/bs14060446, 38920778 PMC11201169

[ref29] Li Z. Xu H. Xu Y. Lu G. Peng Q. Chen J. . (2021). Morinda officinalis oligosaccharides alleviate depressive-like behaviors in post-stroke rats via suppressing NLRP3 inflammasome to inhibit hippocampal inflammation. CNS Neurosci. Ther. 27, 1570–1586. doi: 10.1111/cns.13732, 34559953 PMC8611777

[ref30] Liu Y. Hu Z. Wang J. Liao Y. Shu L. (2022). Puerarin alleviates depressive-like behaviors in high-fat diet-induced diabetic mice via modulating hippocampal GLP-1R/BDNF/TrkB signaling. Nutr. Neurosci. 26, 997–1010. doi: 10.1080/1028415x.2022.2112439 36039913

[ref31] Liu X. Huang R. Wan J. (2023a). Puerarin: a potential natural neuroprotective agent for neurological disorders. Biomed. Pharmacother. 162:114581. doi: 10.1016/j.biopha.2023.114581 36966665

[ref32] Liu T. Li X. Zhang C. Zhao L. Li X. Yu Y. . (2023a). *Lactobacillus* and *Allobaculum* mediates the improvement of vascular endothelial dysfunction during hypertension with TaohongSiwu decoction combined with *Dubosiella newyorkensis*. Heliyon 9:e22572. doi: 10.1016/j.heliyon.2023.e22572, 38089998 PMC10711123

[ref33] Liu T. Su K. Cai W. Ao H. Li M. (2023b). Therapeutic potential of puerarin against cerebral diseases: from bench to bedside. Eur. J. Pharmacol. 953:175695. doi: 10.1016/j.ejphar.2023.175695 36977450

[ref34] Liu X. Sui X. Zhang Y. Yue R. Yin S. (2023b). Efficacy of puerarin in rats with focal cerebral ischemia through modulation of the SIRT1/HIF-1α/VEGF signaling pathway and its effect on synaptic plasticity. Heliyon 9:e15872. doi: 10.1016/j.heliyon.2023.e15872, 37223716 PMC10200855

[ref35] Liu X. Zhang Y. Li W. Zhang B. Yin J. Liuqi S. . (2022). Fucoidan ameliorated dextran sulfate sodium-induced ulcerative colitis by modulating gut microbiota and bile acid metabolism. J. Agric. Food Chem. 70, 14864–14876. doi: 10.1021/acs.jafc.2c06417 36378195

[ref36] Medeiros G. C. Roy D. Kontos N. Beach S. R. (2020). Post-stroke depression: a 2020 updated review. Gen. Hosp. Psychiatry 66, 70–80. doi: 10.1016/j.genhosppsych.2020.06.011, 32717644

[ref37] Mo J. Ni J. Zhang M. Xu Y. Li Y. Karim N. . (2022). Mulberry anthocyanins ameliorate DSS-induced ulcerative colitis by improving intestinal barrier function and modulating gut microbiota. Antioxidants 11:1674. doi: 10.3390/antiox11091674, 36139747 PMC9496020

[ref38] Ozen M. Piloquet H. Schaubeck M. (2023). *Limosilactobacillus fermentum* CECT5716: clinical potential of a probiotic strain isolated from human milk. Nutrients 15:2207. doi: 10.3390/nu15092207, 37432320 PMC10181152

[ref39] Pan Y. Fan F. Jiang J. Zhang Y. (2025). Clinical outcomes of anti-inflammatory therapies inhibiting the NLRP3/IL-1β/IL-6/CRP pathway in coronary artery disease patients: a systemic review and meta-analysis of 37,056 individuals from 32 randomized trials. Inflamm. Res. 74:99. doi: 10.1007/s00011-025-02058-9, 40583093 PMC12206679

[ref40] Shi M. Li Z. Tang Z. Zhou H. Huang X. Wei Y. . (2024). Exploring the pathogenesis and treatment of PSD from the perspective of gut microbiota. Brain Res. Bull. 215:111022. doi: 10.1016/j.brainresbull.2024.111022, 38936669

[ref41] Song X. Wang W. Ding S. Liu X. Wang Y. Ma H. (2021). Puerarin ameliorates depression-like behaviors of with chronic unpredictable mild stress mice by remodeling their gut microbiota. J. Affect. Disord. 290, 353–363. doi: 10.1016/j.jad.2021.04.037 34049088

[ref42] Stolzer I. Scherer E. Süß P. Rothhammer V. Winner B. Neurath M. F. . (2023). Impact of microbiome–brain communication on neuroinflammation and neurodegeneration. Int. J. Mol. Sci. 24:14925. doi: 10.3390/ijms241914925, 37834373 PMC10573483

[ref43] Sun Y. Zhang S. Nie Q. He H. Tan H. Geng F. . (2022). Gut firmicutes: relationship with dietary fiber and role in host homeostasis. Crit. Rev. Food Sci. Nutr. 63, 12073–12088. doi: 10.1080/10408398.2022.2098249 35822206

[ref44] Suñer-Soler R. Maldonado E. Rodrigo-Gil J. Font-Mayolas S. Gras M. Terceño M. . (2024). Sex-related differences in post-stroke anxiety, depression and quality of life in a cohort of smokers. Brain Sci. 14:521. doi: 10.3390/brainsci14060521, 38928522 PMC11201541

[ref45] Tao W. Zhang Y. Wang B. Nie S. Fang L. Xiao J. . (2025). Advances in molecular mechanisms and therapeutic strategies for central nervous system diseases based on gut microbiota imbalance. J. Adv. Res. 69, 261–278. doi: 10.1016/j.jare.2024.03.023 38579985 PMC11954836

[ref46] Tian P. Chen Y. Zhu H. Wang L. Qian X. Zou R. . (2022). *Bifidobacterium breve* CCFM1025 attenuates major depression disorder via regulating gut microbiome and tryptophan metabolism: a randomized clinical trial. Brain Behav. Immun. 100, 233–241. doi: 10.1016/j.bbi.2021.11.023, 34875345

[ref47] Vahid-Ansari F. Lagace D. C. Albert P. R. (2016). Persistent post-stroke depression in mice following unilateral medial prefrontal cortical stroke. Transl. Psychiatry 6:e863. doi: 10.1038/tp.2016.124 27483381 PMC5022078

[ref48] Wu Y. Deng J. Ma J. Chen Y. Hu N. Hao S. . (2024). Unraveling the pathogenesis of post-stroke depression in a hemorrhagic mouse model through frontal lobe circuitry and JAK-STAT signaling. Adv. Sci. 11:2402152. doi: 10.1002/advs.202402152, 38946585 PMC11434213

[ref49] Ye T. Zhang N. Tan X. Zha Q. Chen C. Quan F. . (2025). Electroacupuncture pretreatment alleviates cerebral ischemia-reperfusion-induced intestinal barrier injury and neuroinflammation via inhibition of the TLR4/NLRP3 inflammasome pathway in rats. Brain Res. 1866:149944. doi: 10.1016/j.brainres.2025.149944, 40939710

[ref50] Yuan X. Chen B. Duan Z. Xia Z. Ding Y. Chen T. . (2021). Depression and anxiety in patients with active ulcerative colitis: crosstalk of gut microbiota, metabolomics and proteomics. Gut Microbes 13:1987779. doi: 10.1080/19490976.2021.1987779, 34806521 PMC8632339

[ref51] Zhang Y. Yang Y. Li H. Feng Q. Ge W. Xu X. (2023). Investigating the potential mechanisms and therapeutic targets of inflammatory cytokines in post-stroke depression. Mol. Neurobiol. 61, 132–147. doi: 10.1007/s12035-023-03563-w, 37592185

[ref52] Zheng T. Jiang T. Li R. Zhu Y. Han Q. Wang M. (2024). Circulating interleukins concentrations and post-stroke depression: a systematic review and meta-analysis. Prog. Neuro-Psychopharmacol. Biol. Psychiatry 134:111050. doi: 10.1016/j.pnpbp.2024.111050, 38844127

[ref53] Zhou Y. Huang Y. Bi X. (2026). Targeting the microbiota-gut-brain axis in post-stroke insomnia: a phase-dependent therapeutic framework. Front. Neurosci. 20:1784251. doi: 10.3389/fnins.2026.1784251, 42040336 PMC13102651

[ref54] Zhou H. Wei Y.-J. Xie G.-Y. (2024). Research progress on post-stroke depression. Exp. Neurol. 373:114660. doi: 10.1016/j.expneurol.2023.114660, 38141804

[ref55] Zhou Y. X. Zhang H. Peng C. (2013). Puerarin: a review of pharmacological effects. Phytother. Res. 28, 961–975. doi: 10.1002/ptr.5083, 24339367

[ref56] Zhou Y. Zhao L. Tang Y. Qian S. (2025). Association between serum inflammatory cytokines levels and post-stroke depression among stroke patients: a meta-analysis and systematic review. J. Psychosom. Res. 190:112050. doi: 10.1016/j.jpsychores.2025.112050, 39952012

[ref57] Zou Y. Ding W. Wu Y. Chen T. Ruan Z. (2023). Puerarin alleviates inflammation and pathological damage in colitis mice by regulating metabolism and gut microbiota. Front. Microbiol. 14:1279029. doi: 10.3389/fmicb.2023.1279029, 37908541 PMC10614640

